# Cytomegalovirus infection is common in prostate cancer and antiviral therapies inhibit progression in disease models

**DOI:** 10.1002/1878-0261.70073

**Published:** 2025-06-10

**Authors:** Johanna Classon, Moa Stenudd, Margherita Zamboni, Kanar Alkass, Carl‐Johan Eriksson, Lars Pedersen, Alrik Schörling, Anna Thoss, Anders Bergh, Pernilla Wikström, Hans‐Olov Adami, Henrik Toft Sørensen, Henrik Druid, Jonas Frisén

**Affiliations:** ^1^ Department of Cell and Molecular Biology Karolinska Institutet Solna Sweden; ^2^ Department of Pathology and Oncology Karolinska Institutet Solna Sweden; ^3^ Department of Clinical Epidemiology Aarhus University Hospital Aarhus Denmark; ^4^ Department of Medical Biosciences, Pathology Umeå University Umeå Sweden; ^5^ Department of Medical Epidemiology and Biostatistics Karolinska Institutet Solna Sweden; ^6^ Clinical Effectiveness Group Institute of Health and Society, University of Oslo Oslo Norway

**Keywords:** antiviral treatment, cancer therapy, cytomegalovirus, prostate cancer

## Abstract

Metastatic prostate cancer is incurable, and new therapeutic targets and drugs are urgently needed. Viral infections are associated with several cancer types, but a link between viruses and prostate oncogenesis has not been established. Only recently, an association between human cytomegalovirus (CMV) seropositivity and increased risk of prostate cancer mortality was demonstrated. Here, we show that CMV infection is common in the normal prostate epithelium and in prostate tumor tissue, with 70–92% of tumors being infected. Additionally, we report that commonly studied prostate cancer cell lines are CMV infected. Loss‐of‐function experiments demonstrate that CMV promotes cell survival, proliferation, and androgen receptor signaling, identifying it as a therapeutic target in castration‐sensitive and castration‐resistant prostate cancer. Several anti‐CMV pharmaceutical compounds in clinical use inhibited cell expansion in prostate cancer models both *in vitro* and *in vivo*. We conclude that CMV is common in prostate cancer, promotes core prostate cancer cell programs, and can be inhibited by well‐tolerated drugs. These findings motivate investigation into potential clinical benefits of CMV inhibition in the treatment of prostate cancer.

## Graphical abstract

Human cytomegalovirus infection is common in normal prostate epithelium, prostate tumor tissue, and prostate cancer cell lines. CMV promotes cell survival, proliferation, and androgen receptor signaling. Anti‐CMV pharmaceutical compounds in clinical use inhibited cell expansion in prostate cancer models *in vitro* and *in vivo*, motivating investigation into potential clinical benefits of CMV inhibition in the treatment of prostate cancer.

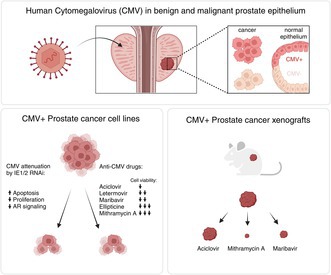

**Abbreviations**
5α‐DHT: 5α‐dihydrotestosterone
AR: Androgen receptor
AR‐FL: Full‐length Androgen receptor
CMV: Human cytomegalovirus
CRPC: Castration‐resistant prostate cancer
CSPC: Castration‐sensitive prostate cancer
FACS: Fluorescence‐activated cell sorting
FISH: Fluorescence *in situ* hybridization
IC50: Half‐maximal inhibitory concentration
IE1/2: Immediate early 1 and 2
IHC: Immunohistochemistry
MBV: Maribavir
MetA: Metastases with high AR activity
MetB: Metastases with a dedifferentiated phenotype
MitA: Mithramycin A
RNAi: RNA interference

## 1 Introduction

Prostate cancer is one of the most common tumor types with more than 350 000 deaths worldwide annually, making prostate cancer one of the leading causes of death from cancer. Only few molecular drivers of lethal prostate cancer that are possible to therapeutically target are known. The most well‐established of these is androgen receptor (AR) signaling, promoting cell proliferation and survival in treatment‐naïve prostate cancer. Androgen deprivation therapy, which has been the standard of care for advanced prostate cancer for decades, suppresses androgen signaling and tumor growth, but the disease ultimately progresses to castration‐resistant prostate cancer (CRPC). Most commonly, restored AR signaling drives growth of CRPC, and next‐generation AR signaling inhibitors such as enzalutamide are initially effective. Treatment resistance can also arise through mechanisms independent of AR, in which AR signaling is low or absent, and this is associated with worse outcome and few treatment options [1, 2]. Although new therapies that prolong life have emerged in the last few years, including poly (ADP‐ribose) polymerase and prostate‐specific membrane antigen targeted therapies, metastatic prostate cancer is still incurable [3] and it is important to identify new actionable therapeutic targets.

Chronic viral infections can induce cancer, alter disease progression, and are potential therapeutic targets. For example, human papillomavirus causes cervical cancer and can be targeted by vaccination to decrease cancer incidence [4, 5], but in most other cancer types of epithelial origin, including prostate cancer, viral infections are not established disease drivers. Furthermore, seropositivity toward known cancer‐associated viruses has not been linked to prostate cancer occurrence. Sporadic reports over several decades describe the potential presence of DNA viruses in prostate cancer but no clear associations identifying them as tumor‐causing agents have been made. Seropositivity toward the herpesvirus human cytomegalovirus (CMV), which is not classified as an oncogenic virus, is not associated with increased risk of prostate cancer incidence [6, 7].

On the other hand, we recently reported that local T‐cell immunity to CMV in HLA‐A*02:01 prostate cancer patients is associated with increased disease recurrence after prostatectomy [8] and found that CMV seropositivity is associated with increased prostate cancer mortality [6]. This suggests that either CMV or an immune response to CMV may promote prostate cancer progression, rather than its initial development. Active CMV can replicate in prostate epithelial cells and cause prostatitis in immunosuppressed patients [9], demonstrating prostate tropism.

CMV is a ~235 kbp large virus with over 150 open reading frames with gene products capable of regulating many cellular processes in order to promote viral propagation and persistence [10]. Although CMV has pro‐cancer properties in cancer models, the majority of studies rely on ectopic expression of CMV genes or analyze outcomes of active lytic infection [11, 12, 13], perhaps not reflecting *in vivo* CMV biology in infected tissues. The majority of humans are persistently infected by CMV, indicated by 83% having antibodies against the virus [14]. This is likely a substantial underestimation of the proportion of the population that is infected, as it is common to detect CMV DNA in seronegative individuals [15, 16, 17, 18, 19, 20, 21, 22], indicating that they are infected but lack a detectable antibody response. Characteristics of latent infection have mainly been studied in blood and bone marrow, as it is well established that CMV persists in hematopoietic lineage cells [21, 23, 24]. Similar to other herpes viruses, CMV can maintain its DNA as episomes that can be replicated and maintained by cellular and viral proteins [23, 25]. Historically, latency has been viewed as a dormant state in which only a restricted set of viral genes were expressed. The modern paradigm defines CMV latency as a state of viral persistence in which viral particles are not being produced but CMV still actively perturb cellular functions and a large set of viral genes are expressed at a very low level [26, 27, 28, 29, 30].

It is, however, notoriously technically challenging to detect endogenous latent infection, which has hampered research on CMV in tissues in health and diseases and led to conflicting data [31]. More than 20 years ago, the benign and cancerous prostate gland was, among other organs and diseases, a suggested site of CMV infection [32, 33, 34], but the validity of this finding has been questioned as other studies have been unable to identify CMV in prostate tissues [35].

We report that CMV infection is common in epithelial cells of the healthy prostate as well as in prostate cancer. We find that commonly studied prostate cancer cell lines are CMV infected, and that CMV promotes cell survival and proliferation, identifying CMV as a therapeutic target in castration‐sensitive and castration‐resistant prostate cancer. We demonstrate that several pharmaceutical compounds used in clinical practice targeting CMV inhibit disease progression in prostate cancer models.

## 2 Materials and methods

### 2.1 Study design and ethical approval of human studies

This study was conducted in accordance with the principles of the Declaration of Helsinki. Presence and characteristics of CMV infection were evaluated in human specimens from several independent cohorts. For analysis of materials from prostate cancer patients, experiments were performed with the understanding and written consent of each subject. Ethical permission for study of human samples from postmortem donors was granted by the regional ethics committee of Sweden in Stockholm (2010/313–31/3). Prostate tissue and blood samples were collected from postmortem donors between 2015 and 2020 through KI Donatum, Karolinska Institutet, Stockholm, Sweden, as described in [6, 8]. Medical history was obtained from medical journals, next of kin, police reports, and patient registry. As per Swedish law, oral informed consent for donation was given by relatives prior to inclusion in the study. The informed consent was documented in writing.

Presence of CMV RNA was evaluated in the prostate cancer cohort CPC‐GENE, in which tumor tissue from patients undergoing radical prostatectomies were collected and RNA‐sequenced [36]. Analysis of sequencing data was permitted by the Swedish Ethical Review Authority (2019–03086, 2020–01374). De‐identified prostate cancer FFPE tissue from prostatectomy specimens was received from the Prostate Cancer Biorepository Network (PCBN) biobank (approval number #15–025). Ethical permission for biobank tissue collection was obtained from the local institutional review board at Memorial Sloan Kettering Cancer Center, New York, USA. A human prostate tissue array (cancer) (NBP2‐30169; Novusbio, Centennial, CO, USA) was used to examine an association between CMV and androgen receptor protein expression. Fresh frozen and FFPE bone metastasis samples from patients with castration‐resistant prostate cancer were collected at Umeå University Hospital (cohort described in, e.g., [37]). Collection and analyses of bone metastases were approved by the local ethics review board of Umeå University (Dnr 2016–332‐32M).

A prospective cohort study based on data from several population‐based Danish linked registries was conducted [38, 39, 40, 41]. Danish law does not require approval from an ethics committee or informed consent from patients to perform registry‐based studies.

### 2.2 Processing of postmortem donor tissue and blood

We collected cross sections of prostate tissue that were designed to include parts of the central‐, transition‐, and peripheral zone of the gland from 41 male postmortem donors aged 19–89 (Fig. S1). One side of the prostate was frozen at −80 °C, and one side was subjected to fixation and paraffin embedding. Serum was separated from other blood products by centrifugation at 20 min, 3500 rpm at 4°. Histological evaluation of prostate cancer was assessed in H&E‐stained FFPE prostate slides by a trained pathologist. An incidental prostate cancer (Gleason score 3 + 3) was detected in a subject, and a suspected incidental prostate cancer was detected in another subject but was difficult to further assess histologically due to postmortal effects on the tissue. The presence of cancer was validated by lack of p63 protein expression by immunohistochemistry. Two additional individuals had been treated for prostate cancer with local radiation or chemotherapy.

Anti‐CMV IgG titer in serum was analyzed using CMV IgG, CMIA assay (Architect, Abbott) or by CMV IgG CLIA (chemiluminescence immunoassay) on the LIAISON®XL Analyzer at Karolinska University Hospital Clinical Microbiology Laboratory.

### 2.3 Characterization of prostate cancer specimens and bone metastases

In material collected in the PCBN, the locations of tumors were outlined in H&E‐stained sections by a pathologist associated with PCBN. Phenotype characterization of bone metastases, divided into MetA (high AR activity), MetB (dedifferentiated, increased proliferation), and MetC (nonluminal, EMT enriched) was described previously [2]. Our bone metastases cohort contained MetA, in this study named AR‐driven, and MetB, in this study named non‐AR‐driven.

Analysis of CPC‐GENE ultra‐deep RNA‐sequencing data has partly been described in [8]. In addition, reads mapping to a CMV genome (NC_006273.2), which was concatenated with the human genome, were explored (*n* = 144). Default parameters with STAR were used to align reads. No CMV‐specific hits were detected when analyzing 173 CMV genes. Contaminant reads mapping to the CMV Major Immediate Early region were recovered in 19 of 144 samples.

### 2.4 Aciclovir population cohort

We conducted a cohort study based on data from the following population‐based Danish registries: the Cancer Registry [38], the Cause of Death Registry, the Danish National Prescription Registry [39], the Danish National Patient Registry [41], and the Danish Civil Registration System [40]. Unambiguous linkage between Danish registries is possible using the Danish Civil Registration Numbers assigned to all Danish residents since 1968, at birth, or upon immigration to the country [40]. Danish citizens have equal tax‐supported access to health care provided by the Danish National Health Service. The Danish Cancer Registry has recorded incident cases of cancer on a nationwide basis since 1943 and has been shown to have an almost complete case ascertainment. Cancer diagnoses in the Cancer Registry are recorded according to the International Classification of Diseases, version 10, and the International Classification of Diseases for oncology for topography and morphology codes. Prostate cancer was defined by ICD‐10 code C61 in the Cancer Registry. Prostate cancer‐specific mortality was recorded in the Cause of Death Registry by ICD‐10 code C61. We retrieved information available on prostate cancer incidence and prostate cancer‐specific mortality from 1970 to 2020.

The Danish National Prescription Registry contains information on all prescriptions dispensed at community pharmacies in Denmark since 1995 [39]. For each prescription, the Prescription Registry records the date and full description of the dispensed product including the Anatomical Therapeutic Code (ATC). We retrieved information available from the Prescription Registry from 1995 to 2020. We identified males prescribed systemic aciclovir (aciclovir ATC J05AB01 or valaciclovir ATC J05AB11) between 1996 and 2020 and used date of first prescription as start of follow‐up (*n* = 313 072). Follow‐up starts one year after the initiation of the Danish National Prescription Registry (1996 and 1995 respectively). Due to the Danish National Prescription Registry not existing prior to 1995, it is unknown if men have been prescribed systemic aciclovir prior to this year. This can result in misclassification, and we partly alleviate this bias by having at least one year look back for each study participant to find actual new drug users and matched nonusers. Systemic aciclovir indications include shingles (varicella zoster reactivation), prophylaxis against genital herpes (herpes simplex 2 reactivation) and prophylaxis against CMV reactivation in patients with transplanted organs or stem cells.

Next, we applied several exclusion criteria: males below age 40 at their first prescription were excluded; *n* = 144 352 (46.1%). Prevalent aciclovir users (determined from 1995 and onwards) were excluded; *n* = 7628 (2.4%). Males prescribed with other nucleotide analogues before the start of follow‐up were excluded; *n* = 460 (0.1%). Males with prostate cancer before the start of follow‐up were excluded; *n* = 4516 (1.4%). Therefore, the final cohort of aciclovir users consisted of 156 116 men. We also required that matched nonusers had no prostate cancer diagnosis and no ATC J05AB (nucleotide analogues including aciclovir and valaciclovir) prescription prior to their index date (the date of first prescription for the corresponding user). Aciclovir users were matched 1:5 to systemic aciclovir/valaciclovir nonusers from the general population (from here defined as aciclovir nonusers). Aciclovir users were matched to nonusers on year of birth and calendar year grouped by four‐year intervals (Table S1). In total, the study cohort included 780 580 aciclovir nonusers (Fig. 4). Calendar year group was used as a matching factor to account for differences in s‐PSA screening with time.

The mean age for aciclovir users was 61.5 (SD, 12.7; Table S1), and study participants were included throughout 1996 to 2020 (calendar year, grouped by 4 years; Table S1). The median follow‐up time was 7.6 years (IQR, 3.5–12.8) with 1 324 582 person‐years for aciclovir nonusers and 6 744 409 person‐years for aciclovir users (Table S1). During follow‐up, 36 428 men (3.9%) were diagnosed with prostate cancer and 8366 men died from prostate cancer (0.9% aciclovir nonusers, 0.8% aciclovir users) (Table S1). Aciclovir users were more likely to have a high Charlson Comorbidity index (three or higher) (Table S1).

### 2.5 Blood CD14 ^+^ enrichment and validation

Peripheral blood from postmortem donors was mixed with PBS‐EDTA (2 mm EDTA) and centrifuged at 900 g for 20 min in a blood separation tube containing 16 mL Lymphoprep™ (STEMCELL Technologies, Vancouver, BC, Canada). White blood cells were separated out, washed with PBS‐EDTA, and run through a 100 μm filter. After 1500 ***g*** for 5 min centrifugation, the pellet was resuspended in 1 mL MACS buffer (PBS‐EDTA, 0.5% (w/w) BSA) and incubated in 1:20 CD14 MicroBeads (Miltenyi Biotec, Bergisch Gladbach, Germany) 20 min at 4 °C. The suspension was washed in MACS buffer, centrifuged, and resuspended in 1 mL MACS buffer. CD14^+^ cells were enriched using LS columns (Miltenyi Biotec) on a MACS® manual separator (Miltenyi Biotec). To validate the bead enrichment, cells were incubated with phycoerythrin (PE)‐conjugated anti‐CD11b monoclonal antibody (1:20, BioLegend, clone ICRF44, San Diego, CA, USA) 30 min at room temperature, centrifuged, and resuspended in MACS buffer. Bead‐enriched cells and nonbead‐enriched cells (flow‐through) were analyzed by flow cytometry on an INFLUX machine. FlowJo version 10.5.3 was used to analyze fluorescence‐activated cell sorting (FACS) plots and provide statistics on CD11b^+^ cell fractions, after gating out cell debris. Bead‐enriched cells were stored in PBS at −80 °C until further analysis.

### 2.6 Immunoblot of human samples and cells

Frozen human prostate tissue was cut in 10–30 μm thin sections on a cryostat into tubes. Lysis buffer (20 mm Tris pH 7,5, 1% triton X‐100, 150 mm NaCl, 5 mm EDTA, 10% glycerol, 10 mm NaF) with Halt protease and phosphatase inhibitor cocktail (78 440; Thermo Fisher, Waltham, MA, USA) was added to samples. Prostate lysates were treated with plastic pestles. Cell lysates and prostate lysates were vortexed for 30 s and were then centrifuged for 10 min at 16,9 RCF at 4 °C, and supernatants were collected. Protein concentration was quantified with Pierce BCA Protein Assay Kit (23 225; Thermo Fisher) according to the manufacturer's instructions. Prostate protein samples (40 μg) and cell protein samples (10–20 μg) were incubated in NuPAGE LDS sample buffer (NP0007; Thermo Fisher) with 10% β‐mercaptoethanol according to manufacturer's instructions. Samples were loaded onto NuPAGE 4 to 12%, Bis‐Tris, 1.5 mm, Mini Protein Gel, 10‐well (NP0335BOX, Thermo Fisher) and electrophoresis was performed in NuPAGE MOPS SDS running buffer (NP0001; Thermo Fisher) with NuPAGE antioxidant (NP0005; Thermo Fisher). BenchMark Pre‐stained Protein Ladder (10 748 010; Thermo Fisher) or PageRuler Prestained protein ladder (26 616; Thermo Fisher) was used as a protein ladder. After electrophoresis, proteins were transferred to Trans‐Blot Turbo Mini 0.2 μm PVDF Transfer Packs (1 704 156, Bio‐Rad, Hercules, CA, USA) using a trans‐blot turbo transfer system (Bio‐Rad). Membranes were incubated in Superblock blocking buffer in TBS (37 535; Thermo Fisher) with 0.05% tween‐20 for a minimum of 30 min or milk for one hour.

Primary antibodies were diluted in blocking buffer and incubated overnight at 4 °C. Primary antibodies used: Cytomegalovirus US28 (1:2000, rabbit, PA5‐102302, Thermo Fisher), Cytomegalovirus UL97 (1:2000, rabbit, PA5‐99784, Thermo Fisher), Androgen Receptor (1:2000, mouse, 411, sc‐7305, Santa Cruz Biotechnology, Dallas, Texas, USA), and Cytomegalovirus IE1/2 (1:2000, mouse, MAB810R, monoclonal, Millipore, Burlington, MA, USA). As an endogenous protein level control, β‐actin (1:5000, mouse, clone AC‐74, A2228, Sigma‐Aldrich, Saint Louis, MO, USA) was used and incubated for one hour. Membranes were washed in TBS with 1% tween‐20 (TBS‐T) and incubated with secondary antibodies diluted in blocking buffer for 2 h (1:5000, anti‐mouse‐HRP, NA931V; anti‐rabbit‐HRP, NA934V, GE Healthcare, Chigaco, Illinois, USA). After washing in TBS‐T, membranes were developed with SuperSignal west dura extended duration substrate (Thermo Fisher) or SuperSignal West Atto (Thermo Fisher) and imaged on a ChemiDoc MP imaging system (Bio‐Rad). Non‐CMV protein blots were developed with ECL and films. Immunoblot bands were quantified in ImageJ64 with a gel analysis tool using β‐actin as a loading control.

### 2.7 CMV IHC on human FFPE tissues

Tissues were fixed in formaldehyde, embedded in paraffin (FFPE), and cut to slides at a thickness of 4 μm. FFPE tissue slides were deparaffinized and rehydrated in xylene and an ethanol gradient. After a wash in dH_2_O, slides were incubated in 1× Antigen Unmasking Solution, Citric Acid Based (100×, H‐3300, Vector Laboratories, Newark, CA, USA) 30 min in a steamer, cooled for 10 min, and then washed in PBS. CMV‐positive control slides (CSC0925P, American MasterTech, Lodi, CA, USA) were incubated in antigen unmasking solution for 20 min in a steamer. Slides were incubated for 10–15 min in BLOXALL blocking solution (SP‐6000, Vector Laboratories) or 3% H_2_O_2_, washed in PBS, incubated for 20 min with FC receptor blocker (Innovex Biosciences, Richmond, CA, USA), incubated for 45 min with 10% donkey serum containing 0,5% triton, and then incubated with avidin/biotin blocking kit (Vector Laboratories) according to manufacturer's instructions.

Primary antibodies were then diluted in 10% donkey serum and incubated at room temperature overnight. Primary antibodies used: Cytomegalovirus US28 (rabbit, 1:150, PA5‐39864, polyclonal, Thermo Fisher), Cytomegalovirus pp65 (mouse, 1:50, clone CH12, sc‐56 976, monoclonal, Santa Cruz Biotechnology), Cytomegalovirus pp71 (goat, 1:200, clone vC‐20, sc‐33 323, polyclonal, Santa Cruz Biotechnology), Cytomegalovirus IE1 (mouse, 1:10, pp72, clone 6E1, sc‐69 834, Santa Cruz Biotechnology), Cytomegalovirus IE1/2 (mouse, 1:150, MAB810R, monoclonal, Millipore), Chromogranin A (mouse, 1:100, LK2H10, MA5‐13096, Thermo Fisher), keratin 5 (rabbit, 1:200, EP1601Y, ab52635, Abcam), keratin 18 (rabbit, 1:100, H‐80, sc‐28 264, Santa Cruz Biotechnology), TP63 (mouse, 1:100, clone 4A4, CM163A, Biocare Medical, Pacheco, CA, USA), Ki‐67 (rabbit, 1:100–250, clone SP6, Thermo Fisher), wide spectrum cytokeratin (rabbit, 1:100, ab9377, abcam, Cambridge, UK), pan‐keratin (mouse, 1:200, clone C11, #4545, Cell Signaling, Danvers, MA, USA), and AR (rabbit, 1:50–100, clone SP107, Thermo Fisher).

After washing in PBS, slides were incubated with a secondary antibody conjugated with biotin (donkey anti‐mouse biotin or donkey anti‐rabbit biotin, 1:500, Jackson laboratories, Bar Harbor, Maine, USA) or a secondary antibody conjugated with a fluorophore (donkey anti‐mouse 488/cy3/cy5, donkey anti‐rabbit 488/cy3/cy5, Jackson laboratories). If incubated with a secondary antibody conjugated with biotin, slides were then incubated with VECTASTAIN Elite ABC HRP Reagent, R.T.U. (PK‐7100, Vector laboratories) 30 min, after which slides were washed in PBS and incubated with ImmPACT DAB (Vector Laboratories) or TSA using the Alexa Fluor 488 Tyramide SuperBoost kit (B40932, Thermo Fisher) according to the manufacturer's instructions. For co‐labelling, slides were treated with 3% hydrogen peroxide for 15 min, incubated with the primary antibody overnight, and was then developed with TSA Tyramide 555 or Tyramide 647. DAB‐stained slides were counterstained with hematoxylin QS (Vector Laboratories) after washing in water, dehydrated in an ethanol series from 50% to 100%, incubated in xylene, air dried, and mounted with pertex mounting medium (Histolab). Fluorescently stained slides were counterstained with DAPI (1:5000, Sigma‐Aldrich) and mounted with Prolong Gold Antifade mounting medium (Thermo Fisher).

### 2.8 CMV DNA *in situ* hybridization

FFPE prostate tissue slides were deparaffinized and rehydrated in xylene and ethanol gradient. Animals were perfused with 4% formaldehyde and were postfixed overnight. The prostate glands and prostate cancer xenografts were dissected and incubated in sucrose 30% w/w at 4 °C. Tissue was placed in OCT and cut to glass slides on a cryostat at 12 μm thickness. Tissue slides were stored at −20 °C. Slides were warmed at 42 °C for 1 h. After a wash in dH_2_O, FFPE slides were incubated in 1× RISH Retrieval (RI0209M, Biocare Medical) 15 min and were thereafter cooled for 10 min. All slides were incubated with 3% H_2_O_2_ in 30% methanol for 5 min and washed in dH_2_O. They were then treated with RISHzyme (1:4 FFPE prostate tissue; 1:4 mouse prostate OCT; 1:8 xenografts OCT) in RISHzyme buffer (Biocare Medical) for 1 min. Slides were then incubated with RNAse, DNAse free (Roche, Sigma‐Aldrich) 1:5 in 2× SSC buffer at 37 °C for 30 min. RNAse was washed off and slides were incubated with a digoxigenin labeled RISH CMV probe (R10011T, Biocare Medical) at 95 °C for 15 min (FFPE prostate tissue) or 5 min (mouse prostate and xenografts) and then at 37 °C overnight.

Hybridized slides were then subjected to stringency washes. Slides were washed in 4× SSC 5 min ×2 at room temperature and 0.01× SSC 5 min ×2 80 °C. Slides were incubated with secondary reagent and tertiary reagent per protocol (RISH HRP detection kit, Biocare Medical) after which CMV hybridization was developed with TSA coupled to Cy3 (NEL744001KT, PerkinElmer, Waltham, MA, USA) or using Alexa Fluor 488 tyramide SuperBoost kit (B40932, Thermo Fisher) according to manufacturer's instructions. For co‐labelling of FFPE prostate slides were treated with 3% hydrogen peroxide for 15 min. After washing in PBS, slides were stained as described for IHC. FFPE IHC staining was developed with TSA using Tyramide 555 or Tyramide 647 or with DAB (as described for IHC). For other tissues, slides were incubated with 10% NDS containing 0.5% triton 45 min after which slides were incubated overnight with an antibody against wide spectrum cytokeratin (1:100, rabbit, ab9377, abcam) diluted in 10% NDS. After washing in PBS, slides were incubated with a secondary antibody 1 h (donkey anti‐rabbit‐488, 1:500, Jackson Laboratories), after which slides were stained with DAPI (1:5000, sigma) and mounted with Prolong Gold mounting medium (ProLong™ Gold Antifade Mountant, Thermo Fisher).

### 2.9 Immunofluorescence on cells and mouse tissue

Cells grown in 8‐well culture slides (BD Bioscience, Milpitas, CA, USA) were fixed in 4% formaldehyde 10–15 min and washed in PBS. Mice were perfused with 4% formaldehyde overnight and then washed in PBS. Xenografts and tissues were incubated in 30% sucrose and then embedded in OCT and cut at 12 μm thin sections to glass slides in a cryostat. Cells were incubated with 3% BSA with 0.3% triton or 10% Donkey serum with 0.5% triton for 20 min at room temperature. Tissues and xenografts were incubated with 10% Donkey serum with 0.5% triton 45 min. Primary antibodies were diluted in either 1% BSA or 10% donkey serum and incubated at room temperature for 3 h or overnight at 4 °C.

Primary antibodies used: γ‐H2AX (pS139) (rabbit, 1:500, ab2893, Abcam), Cleaved caspase‐3 (rabbit, 1:250, clone D175, 9661S, Cell Signaling), Histone H3 (phospho S10) (rabbit, 1:500–1000, ab5176, Abcam), Ki‐67 (rabbit, 1:250, clone SP6, Thermo Fisher), Ki‐67 (rat, 1:500–1000, clone SolA15, 14–5698‐82, eBioscience, San Diego, CA, USA), Cytomegalovirus IE1/2 (mouse, 1:200, MAB810R, Millipore), Cytomegalovirus UL97 (rabbit, 1:100, gift from the Coen laboratory, Harvard University), SP1 (rabbit, 1:100, 5931S Cell Signaling), TOPIIB (rabbit, 1:100, ab72334, Abcam), and pRB (S807/811) (rabbit, 1:250, D20B12, 8516, Cell Signaling).

Slides were then washed with PBS and incubated with secondary antibody (Donkey anti‐rabbit cy3, 1:1000, Jackson Laboratories), diluted in 1% BSA for 45–60 min. For EdU detection, Click‐it EdU Alexa Fluor 647 Imaging Kit or Click‐it Edu Plus Alexa Fluor 647 Imaging Kit (Thermo Fisher ) was used prior to antibody staining. Slides were washed with PBS and incubated with DAPI (1:5000, Sigma‐Aldrich), washed in PBS, and mounted with ProLong Gold Antifade Mountant (Thermo Fisher).

### 2.10 Microscopy and image analyses

CMV IHC and *in situ* hybridization on human prostate tissue visualized with DAB was analyzed using a light microscope (CTR6000, Leica, Wetzlar, Germany), and pictures were taken using LAS X software (Leica) with a 40× or 20× objective. CMV IHC and *in situ* hybridization visualized with fluorescence was analyzed using a Zeiss Imager Z2 or a Zeiss LSM 700 confocal microscope. Pictures were taken using ZEN 2012 SP1 (8.1) software (Zeiss, Oberkochen, Germany) and images for publication were processed in ImageJ64 or imagej v.1.52 and photoshop. When analyzed in fluorescence microscope, pan‐keratin was used as a marker to define epithelial cells from other cell types.

For quantifications of CMV IHC abundance, a 20× objective was used to analyze a whole section of prostate tissue and a 10× objective was used in analyses of bone metastases. When an area with epithelium was in sight, this was determined to be positive or negative for CMV‐containing epithelial cells. A CMV‐positive epithelial area could be completely positive for CMV or contain a lower number of CMV‐positive cells. When analyzed in light microscope, no marker for epithelial cells was used, as the epithelium was clearly visible with hematoxylin. For CMV‐US28, varying numbers of cells with very high US28 cytoplasmic staining were found throughout tissue sections, independent of CMV serostatus. These were considered as background‐stained cells and were not considered when determining CMV abundance in epithelial areas.

Intensity in cell nuclei (CMV DNA, AR protein) was compared in confocal images with ImageJ64 or imagej v.1.52 using the IntDen function. For quantification of the percentage of cells positive for a marker, the number of cells was either counted manually or by using a cell quantification feature in imagej using threshold to quantify number of cells. For quantification of number of γH2AX foci per cell, 27–50 cells were analyzed per well in ImageJ64 in images taken with a 40× or 63× objective.

### 2.11 DNA extraction and quantitative CMV PCR

Frozen tissue was cut into DNase‐free Eppendorf tubes in a clean cryostat in which the knife was changed and cryostat was cleaned with ethanol between each specimen, in order to reduce the risk of contamination. Tissues and cell pellets were stored at −80 °C until DNA extraction. DNA was extracted using the DNeasy Blood and Tissue Kit (QIAGEN, Hilden, Germany) using manufacturer's instructions including treatment of samples with RNAse A (QIAGEN). DNA concentration was measured on a nanodrop or on Qubit using Qubit dsDNA BR Assay Kit (Thermo Fisher) according to manufacturer's instructions.

Amplirun cytomegalovirus DNA control (MBCO16, Vircell, Granada, Spain) was used as a positive control. Positive control DNA was reconstituted in 100 μL buffer as per the manufacturer's instructions and diluted 1:10 in dH2O. Of this, 1 μL was used per qPCR reaction using TaqMan fast master mix (Thermo Fisher) and TaqMan primer/probes described below. The restriction enzyme *BsrI* has 387 restriction sites in the CMV strain Merlin. 1000 ng prostate DNA or 300–500 ng bone metastasis DNA was incubated in a 25 μL reaction containing 1× NEBuffer 3.1 (NEB, Ipswich, MA, USA) with or without 0.2 μL of the restriction enzyme Bsr1 (10 000 units·mL^−1^; NEB) at 65° for 16 h and then 85° for 20 min. For samples treated with bsr1, 5 μL was loaded into one qPCR reaction. Otherwise, the input amount of DNA was 500 ng per reaction for cell lines and postmortem prostate DNA samples and 100–200 ng for CD14^+^ cells. Samples were considered positive if a successful qPCR amplification curve was produced. For cell lines in experiments, 10 ng DNA was used. qPCR was performed in 96‐well plates and run on a 7500 Fast Real‐Time PCR system (Applied Biosystems, Waltham, MA, USA) or CFX96 system (Bio‐Rad) with 60–70 cycles. The following master mixes were used: TaqMan fast master mix (Thermo Fisher); UL83, GoTaq Probe qPCR master mix (Promega); UL83, UL37, and qRT‐PCR Brilliant III Probe Master Mix with ROX (Agilent); UL32, UL36, UL38, UL87, UL97, UL122. The predesigned TaqMan primer/probe GAPDH (Hs02786624_g1) was used. Several custom TaqMan primer/probes were used. See Table S3 for primer/probe sequences.

Size of PCR products was visualized on a 3% agarose gel with a 50 bp ladder. The correct identity of PCR products in prostate tissues was validated with Sanger sequencing on PCR products cloned into a plasmid with the TOPO TA Cloning Kit (K457501, Thermo Fisher).

### 2.12 RNA extraction and RT‐qPCR

For examination of CMV gene expression in cell lines, RNA was extracted with RNeasy Plus Mini kit (74 034, QIAGEN). Prior to cDNA synthesis, RNA was treated with ezDNAse enzyme (Thermo Fisher) according to the manufacturer's instructions. cDNA synthesis was performed with SuperScript IV First‐Strand Synthesis System (Thermo Fisher) using 5 μg RNA input with either oligo DT or random hexamers at 50 or 65 °C. Of the cDNA library, 2 μL was used as input for each RT‐qPCR reaction. RT‐qPCR was performed using Premix Ex Taq (Probe qPCR) (RR390L; TAKARA; Kusatsu, Japan) on a CFX96 Real‐Time System (Bio‐Rad) qPCR machine. Custom TaqMan primer/probes to detect LUNA, UL97, and UL122–UL123 were used.

For cell experiments, RNA was extracted with RNeasy Plus Mini kit (74 034, QIAGEN) and cDNA was made from RNA with SuperScript VILO cDNA synthesis kit (11 754 050, Thermo Fisher). RT‐qPCR was performed using TaqMan Fast Advanced Master Mix (4 444 556, Thermo Fisher) and TaqMan primer/probes. RT‐qPCR was performed on a 7500 Fast Real‐Time PCR System (Thermo Fisher) or a CFX96 Real‐Time System (Bio‐Rad). These TaqMan primer/probes were used: AR (Hs00171172_m1), KLK3 (Hs02576345_m1), TMPRSS2 (Hs01122322_m1), and GAPDH (Hs02786624_g1). AR‐V7 TaqMan primer/probes were custom made, see Table S4 for primer/probe sequences.

### 2.13 Cell line culture conditions

LNCaP (RRID:CVCL_0395), DU145 (RRID:CVCL_0105), and PC3 (RRID:CVCL_0035) were purchased from ATCC and cultivated in RPMI‐1600 (Thermo Fisher) with 10% FBS and 1% Penicillin–Streptomycin (PS). VCaP (RRID:CVCL_2235) and MyC‐CaP (RRID: CVCL_J703) were purchased from ATCC and cultivated in DMEM (Thermo Fisher) with 10% FBS and 1% PS. WI‐38 (RRID:CVCL_0579) was purchased from ATCC and cultivated in DMEM with GlutaMax (Thermo Fisher) with 10% FBS and 1% PS. LAPC‐4 (RRID:CVCL_4744) was a gift from Robert Reiter, University of California, Los Angeles, USA. LAPC‐4 was cultivated in IMDM with 5% FBS and 1% PS. LREX’ (RRID:CVCL_UD76) [42] was a gift from Charles Sawyers, MSKCC, New York, USA. LREX’ was cultivated in 20% FBS, and 1 μM enzalutamide and 1% PS. LNCaP‐abl (RRID:CVCL_4793) [43] was a gift from Helmut Klocker, Medical University of Innsbruck, Austria. LNCaP‐abl was cultivated in RPMI‐1600 with 10% charcoal‐stripped FBS and 1% PS. Since their arrival to our laboratory, the cell lines have been handled and cultured in the absence of purified CMV, minimizing risk of contamination from laboratory strains. All experiments were performed with mycoplasma‐free cells. Cell lines were authenticated by Eurofins Genomics Cell Line Authentication Service (Ebersberg, Germany) using AmpFlSTR Identifiler Plus PCR Amplification Kit (Thermo Fisher) within the last 3 years.

### 2.14 Prostate cancer cell transfection

Cells were treated with RNA interference with Lipofectamine RNAiMax transfection reagent (Cat. No. 13778030, Thermo Fisher) according to the manufacturer's instructions. For stealth siRNA, stock solutions of siRNA used at 20 μm, and for silencer select siRNA, stock solutions of siRNA at 10 μm were used. As a control, scrambled siRNA was used: Stealth RNAi siRNA Negative Control, Med GC (Thermo Fisher) or Silencer Select Negative Control #1 siRNA (Thermo Fisher). Stealth siRNA against SP1 and TOPIIB was used. Custom‐designed Stealth siRNA and Silencer Select siRNA (Thermo) were used to target CMV genes. Silencer Select siRNA was used against CMV‐IE1/2 (2) with sequences used from [44]. Sequences of siRNA are shown in Table S2.

### 2.15 Prostate cancer cell treatment with compounds

Following drugs were administered to cell lines *in vitro*: 5‐α DHT (5α‐Androstane‐17β‐ol‐3‐one; A8380, Sigma‐Aldrich), Aciclovir Hospira, concentrate for intravenous infusions (25 mg·mL^−1^ aciclovir (111 mm), sodium hydroxide 4,6 mg·mL^−1^, pH 11.3–11.5, Hospira, Pfizer), Cisplatin (2251; Tocris, Bristol, UK), Ellipticine (sc200878; Chemcruz, Santa Cruz Biotechnology), Etoposide (E1383; Sigma‐Aldrich), Enzalutamide (Santa Cruz Biotechnology), Ganciclovir (Sigma‐Aldrich), Letermovir (Cayman chemical, Ann Arbor, Michigan, USA), Maribavir (MedChemTronica), and Mithramycin A (sc‐200 909; Santa Cruz Biotechnology). EdU (Thermo Fisher) was administered 1 h prior to cell fixation at a concentration of 10 μm.

### 2.16 Cell viability and apoptosis assays

Cells were seeded in replicate or triplicate. Cell viability was measured by CellTiter‐Glo 2.0 Cell Viability Assay (G9242, Promega, Madison, Wisconsin, USA) according to the manufacturer's instructions on a luminescence reader. Relative IC50 was fitted with a nonlinear fit and calculated in GraphPad Prism 8. Similarly, absolute IC50 was calculated, with 0% as baseline and 100% as top restraints. For the analysis of apoptosis, Caspase‐Glo 3/7 Assay System (G8090, Promega) was used. Apoptosis induction was determined by dividing apoptosis luminescence with cell viability luminescence. The resulting values were then compared between treated and control cells to determine the fold change in apoptosis.

### 2.17 Animal experiments

Animal experiments were approved by the Swedish Board of Agriculture, Stockholm (application 6727–18, application N132/13 with amendments N150/16 and N170/16). Male SCID mice 8 weeks or older (Fox Chase SCID® Mice CB17/Icr‐Prkdcscid/IcrIcoCrl, Charles River Laboratories) were used. Animals were kept in standard housing conditions with a 12:12 h light:dark cycle. All animals had unrestricted access to food and water. DU145 and PC3 cells were implanted subcutaneously. When the size of xenografts had developed to a mean 150 mm^3^ in volume, drug treatment was initiated. Animals were administered with 1) vehicle or mithramycin A (750 μg·kg^−1^), 2) vehicle or maribavir (100 mg·kg^−1^), 3) control or valaciclovir (25 mg·g food^−1^) and tumor volumes were evaluated weekly. The endpoint of the experiment was tumor size 1000 mm^3^ or when the humane endpoint was reached.

To determine dosage of aciclovir and plasma concentrations, adult male animals were administered with valaciclovir in food, after which blood was taken and aciclovir concentrations were measured. Valaciclovir (VALTREX, GlaxoSmithKline) 500 mg caplets were grinded to a fine powder using pestle and mortar. Valaciclovir powder was mixed into 60–80 grams of porridge food sucrose. Concentrations of valaciclovir were 25 mg valaciclovir·g food^−1^. After seven – nine days, blood was drawn cardially peri‐mortem during deep chloral hydrate or pentobarbital sedation. Blood was let to coagulate and was then centrifuged for 10 min at 1500 *g*. Serum was transferred to a 1.5 mL polypropylene tube and stored at −20 °C until analysis. Serum aciclovir concentration was measured using LC–MS/MS (routinely used to measure aciclovir levels in clinical serum samples), performed at the Clinical Pharmacology Laboratory, Karolinska University Hospital, Huddinge, Sweden.

Male SCID mice were implanted with 1.5 × 10^6^ PC3 cells or 2.0 × 10^6^ DU145 cells subcutaneously on the lower dorsal flank in 1:1 RPMI medium:matrigel (Corning Matrigel Matrix, phenol red free, Corning, Corning, NY USA), in a total volume of 100 μL. Tumors were measured manually with calipers using the formula (x*y^2^)/2. X and y are two width measurements with x being the largest measured value. When tumors had developed to a mean 150 mm^3^ in volume, animals were administered with drugs, after which animals were weighed and monitored for tumor growth weekly. Mithramycin A (750 μg·kg^−1^) was administered i.p. once a day in week 1. In weeks 2–3, mithramycin A was administered four times with at least one‐day intermission in between dosages. In week 4, mithramycin A was administered daily. In weeks 5–6, the same schedule as week 2‐3 was followed. In week 7, mithramycin A was administered daily. Valaciclovir (25 mg·g food^−1^) was given in porridge food in intervals of 5 days with 7‐day intermission. The endpoint of the experiment was a tumor size of 1000 mm^3^ or when a humane endpoint was reached. Maribavir (100 mg·kg^−1^) was given via oral gavage twice per day every weekday.

In the aciclovir xenograft experiment, deep, intradermal tumors and a tumor that was much larger at the endpoint than anticipated by manual tumor measurements were excluded from the analyses.

### 2.18 Statistical analyses

All statistical analyses were performed in GraphPad prism 8.0. All plots were also made in GraphPad prism 8.0. Two‐sided unpaired t‐tests were performed on numerical continuous data with a normal distribution. Groups with non‐normal numerical continuous data were analyzed with the Mann–Whitney test. Correlation analyses on non‐normal distributed data were performed with Spearman correlation analyses, and correlation analyses on normal distributed data were performed with Pearson correlation analyses and with linear regression when suited. Paired/matched values were analyzed with a two‐sided paired t‐test. Values that were compared to a fixed value (e.g., 100% cell viability in controls) were statistically evaluated for statistical deviation from this value with one‐sample t‐test. Proportions between two groups were analyzed with Fisher's exact test. Non‐normal distributed data with more than two groups were compared with Kruskal–Wallis multiple comparisons.

Survival analysis with log‐rank test for animal survival in xenograft experiments was performed with log‐rank test (Mantel–Cox). Tumor size over time in mouse experiments was compared between treatment groups with repeated measures mixed‐effect models, that allows for missing values, with multiple comparisons for each time point. Statistical analyses of the aciclovir population cohort were performed using SAS 9.4 software (SAS Institute Inc., Cary, USA). In a Cox proportional hazard model, aciclovir usage, age at index date, calendar year, and Charlson Comorbidity index were included as variables.

## 3 Results

### 3.1 CMV in prostate epithelial cells

**Fig. 1:**
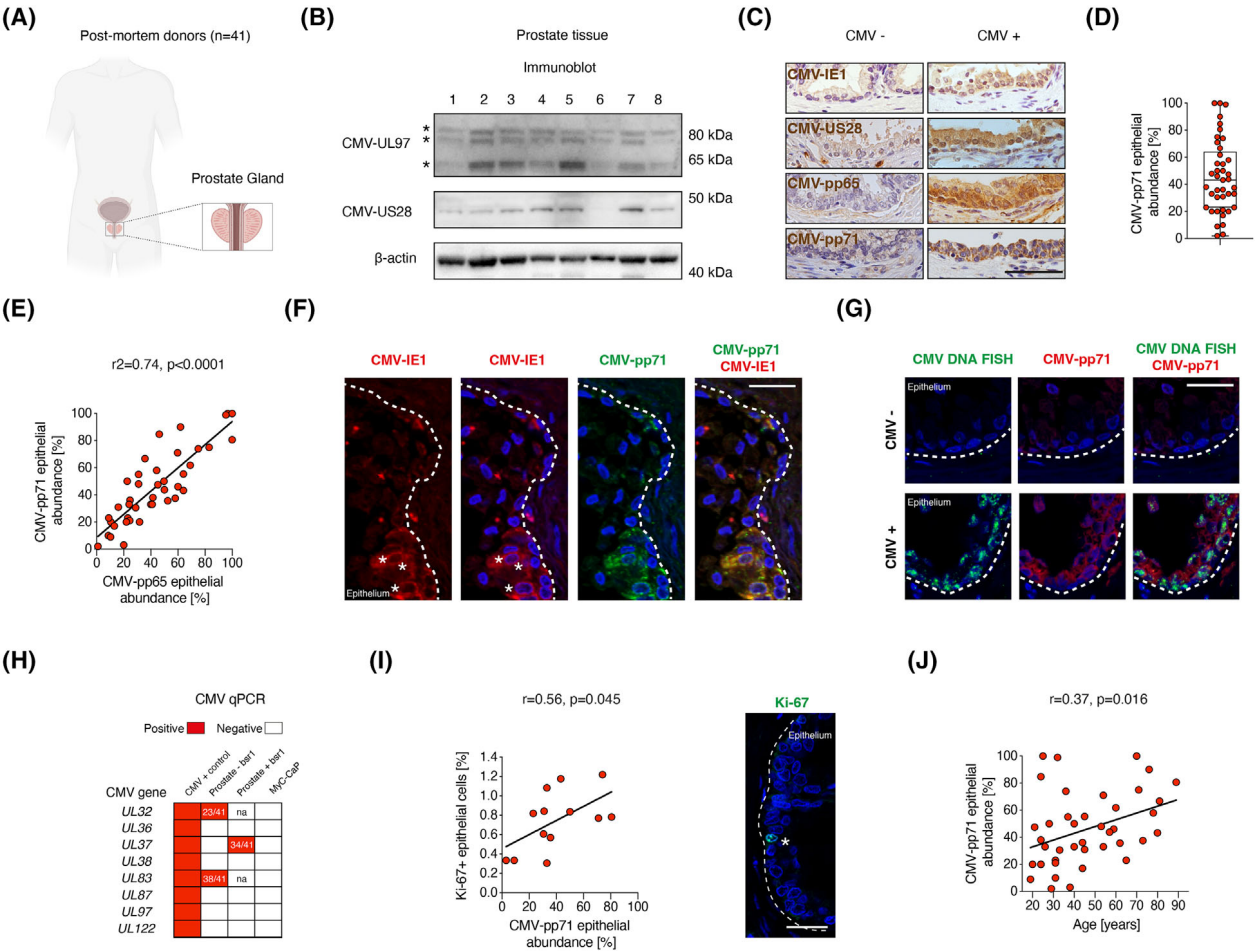
CMV in prostate epithelial cells. (A) Illustration, made with Biorender.com, of prostate collection from postmortem donors. (B) Immunoblot of prostate tissue homogenates for the proteins CMV‐UL97 (with three bands as expected [81], marked with asterisks), CMV‐US28, and β‐actin from eight men. kDa is kilodalton. (C) Images of prostate sections with CMV antibody staining in brown in the epithelium. Nuclei are labeled in purple with hematoxylin. Scale bar 50 μm. (D) Quantification of CMV‐pp71 abundance in epithelial cells. All data points (*n* = 41) are shown in the graph and summarized in the box plot (median, 25–75th percentiles, error bars show min‐max values). (E) Linear regression comparing CMV‐pp71 and CMV‐pp65 abundance in the prostate epithelium (*n* = 41). (F) Co‐labelling of CMV‐pp71 (green) and CMV‐IE1 (red). Scale bar 25 μm. Asterisks label cell nuclei with partial CMV‐IE1 protein expression. (G) Co‐labelling of CMV DNA (green) and CMV‐pp71 (red). Scale bar 25 μm. (H) CMV qPCR heatmap. CMV qPCR assays detected CMV DNA in the positive control (purified CMV DNA) but not in the negative control (mouse prostate cancer cell line MyC‐CaP). Numbers denote how many prostates were positive and evaluated with qPCR with or without *Bsr1* restriction enzyme pretreatment. Three prostate samples were evaluated in the screening of qPCR assays. Na is not analyzed. (I) Pearson correlation of the percentage of Ki‐67^+^ cells and CMV‐pp71 abundance (*n* = 13). Labeling with Ki‐67 (green). Star points out a Ki‐67^+^ epithelial cell. Scale bar: 25 μm. (J) Pearson correlation between CMV‐pp71 epithelial abundance and age (*n* = 41). In (F), (G) and (I), nuclei are labeled in blue with DAPI and dotted lines depict the basal lamina of the epithelium. CMV, Human cytomegalovirus.

We set out to characterize to what extent CMV is present in healthy prostate tissue and in prostate cancer using several orthogonal methods. CMV gene expression is often difficult or not possible to detect in endogenous latent infection, in contrast to active lytic infection, although CMV DNA is present in both viral states [26, 29]. CMV target enriched sequencing of cells from blood and *in vitro* models of CMV latency has shown very low expression of genes from across the CMV genome [26]. Protein expression from a plethora of CMV genes is therefore expected, but has to our knowledge not been explored, in endogenous latent infection of hematopoietic cells. In models of latency, CMV protein expression is much reduced compared to lytic infection but even so, they can have essential functions in viral maintenance [25, 45, 46]. CMV gene expression has not been detected by RNA sequencing of prostate samples [29, 47, 48] and we failed to detect CMV transcripts in an analysis of deeply sequenced prostate cancer (see Methods S1). This null‐finding proposed that CMV was either absent, scarce, or latent. To assess if a latent CMV infection could be present in the prostate gland, we collected prostate tissue postmortem from 41 men aged 19–89 years of whom two men had been diagnosed with prostate cancer prior to death (Fig. 1A, Fig. S1A). Presence of CMV DNA and proteins were analyzed. First, we identified the well‐characterized CMV proteins UL97 and US28, both expressed during hematopoietic latency [26], in prostate tissue homogenates by immunoblot (*n* = 8; Fig. 1B). CMV protein levels did not correlate with postmortem interval (Fig. S1B, *n* = 7).

To localize and quantify the abundance of CMV, we used immunohistochemistry (IHC) and focused on epithelial cells since they are the cells of origin of prostate cancer and show CMV tropism [9]. We studied the CMV proteins IE1, US28, pp65, and pp71, of which all are expressed during latency [26] and IE1, US28, and pp71 have well‐established latency‐associated functions [25, 45, 46]. All four CMV IHC assays identified CMV in positive controls (Fig. S1C), and no signal was detected in negative control assays (Fig. S1D). The CMV proteins IE1, US28, pp65, and pp71 were all detected in epithelial areas (Fig. 1C,D) with high concordance (Fig. 1E; Fig. S1A,E). Different CMV proteins were codetected in the same prostate epithelial cells, and CMV protein expression was not exclusive to a specific cellular location (Fig. 1F; Fig. S1F). CMV was detected in the prostate in all subjects, with the proportion of the prostate epithelium being infected ranging from 2% to 100% with a mean of 46% (*n* = 41, Fig. 1D). CMV DNA (*RNA2.7* gene) was detected in tissue sections by *in situ* hybridization and was primarily found in cell nuclei and localized to CMV protein‐positive epithelial areas (Fig. 1G), corroborating that CMV IHC specifically identifies CMV in prostate epithelial cells.

Although CMV DNA is easily detected by PCR during active lytic infection, it is more challenging to capture when the virus is latent [24, 49], perhaps related to a complex circular viral DNA conformations during latency. Of eight CMV qPCR assays that readily identified purified CMV DNA, two (CMV‐*UL32*, CMV‐*UL83*) identified CMV in prostates, confirmed by sequencing of PCR products (Fig. 1H; Fig. S1G). These also identified CMV DNA in hematopoietic lineage cells, with the highest quantities in CMV IgG^+^ donors (Fig. S1H–K), in line with previous reports [50, 51, 52], supporting the specificity of the assays. CMV‐*UL37* was detected in prostates after pretreatment of DNA with a restriction enzyme (Fig. 1H), suggesting that linearized shorter fragments of viral DNA may be more accessible for PCR amplification. The only prostate in this cohort that was negative in all qPCR assays had the lowest CMV epithelial abundance (2%) by IHC (Fig. 1D,H; Fig. S1A).

Within CMV^+^ epithelial areas, either patches of cells or whole areas of glandular structures were infected (Fig. 1C), which included one or more of the main epithelial subtypes basal‐, luminal‐, and neuroendocrine cells (Fig. S1L). CMV abundance correlated with the number of epithelial cells that were positive for the proliferation marker Ki‐67 (*r* = 0.56, *P* = 0.045, Fig. 1I) and the proportion of CMV‐infected epithelial cells increased with age (r = 0.37, *P* = 0.016, Fig. 1J), possibly reflecting the expansion of CMV‐infected cells with time.

In summary, we identified CMV infection in epithelial cells of the prostate gland by immunoblot, IHC, DNA *in situ* hybridization, and PCR. Different CMV proteins colocalized with each other and with CMV DNA, with CMV^+^ epithelial clones taking over a larger area of the prostate with time.

### 3.2 CMV in prostate cancer

**Fig. 2:**
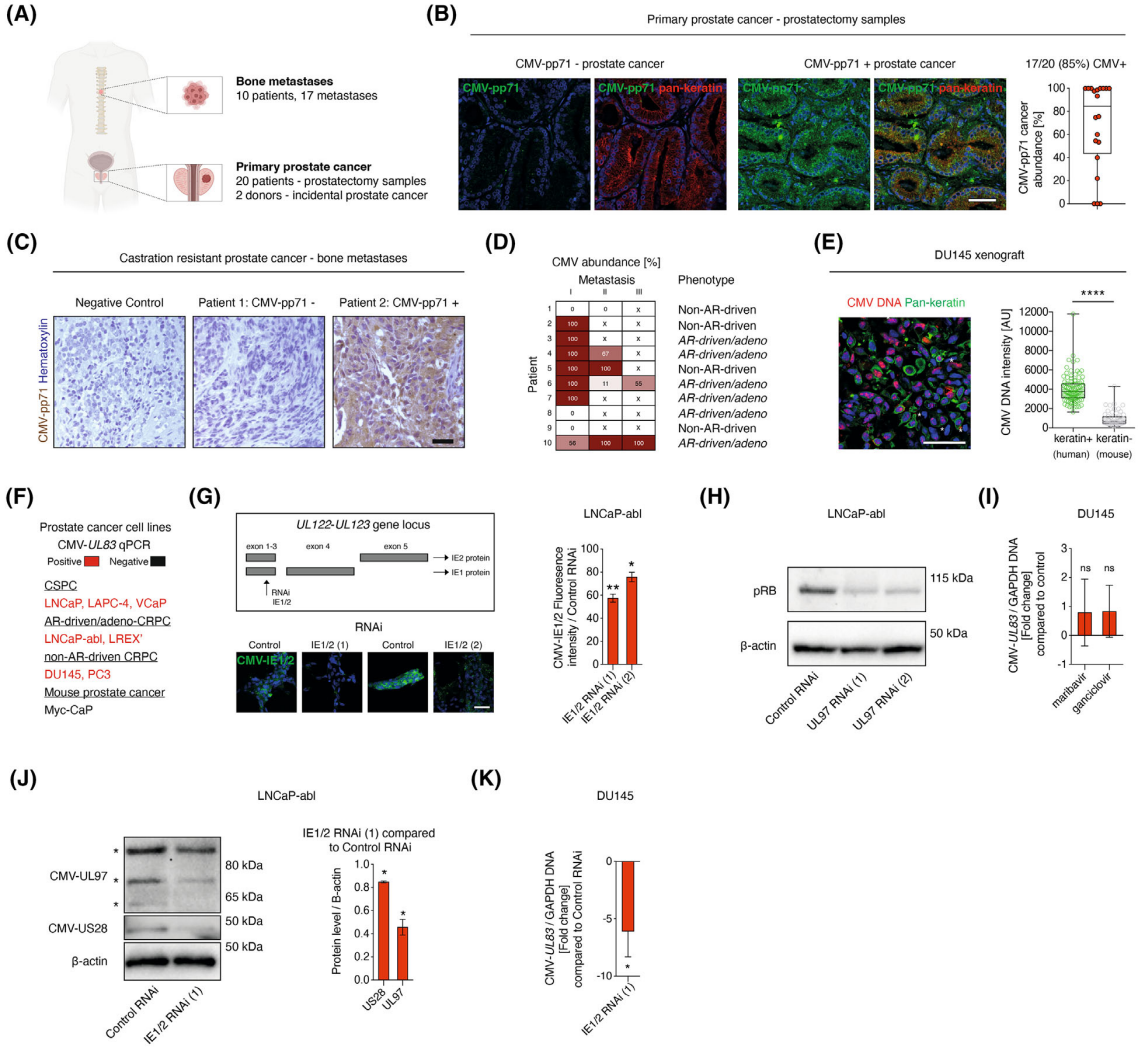
CMV in prostate cancer and prostate cancer cell lines. (A) Illustration, made with Biorender.com, of prostate cancer cohorts. (B) Abundance of CMV‐pp71 (green, %) in tumors (*n* = 20), determined by IHC. Pan‐keratin (red) was used as a marker for benign and malignant epithelial cells. Prostate cancer was identified histologically in pathologist‐outlined areas. Scale bar: 25 μm. (C, D) Bone metastases from 10 CRPC patients were examined for CMV‐pp71 protein expression with IHC. Nuclei are labeled in purple with hematoxylin. Scale bar: 50 μm. Phenotypes were defined as AR‐driven (MetA, high AR activity) and non‐AR‐driven (MetB, dedifferentiated phenotype) [2]. In five patients, more than one metastasis was examined, labeled metastases I, II, and III, and percentage of CMV‐pp71+ areas are shown in heat map. x means no metastasis to analyze. (E) CMV DNA FISH (red) in DU145 xenografts (*n* = 3). Fluorescence intensity in cell nuclei in pan‐keratin^+^ (epithelial cells, here labeling human cells, green) and pan‐keratin^−^ cells (here labeling mouse cells) were compared with unpaired *t*‐test. AU, arbitrary unit. Scale bar: 50 μm. All datapoints (all cells analyzed) are shown in plot. (F) qPCR of CMV‐*UL83* in cell lines. (G) LNCaP‐abl transfected with IE1/2 RNAi (1) or IE1/2 RNAi (2), targeting the *UL122‐UL123* exon 3 (illustration), reduced mean IE1/2 fluorescence (green) compared to Control RNAi, analyzed with one‐sample *t*‐test. Scale bar: 50 μm. *n* = 3. (H) Ser^807^/Ser^811^ pRB and β‐actin expression was examined by immunoblot (*n* = 3). (I) DU145 treated with vehicle, 30 μm maribavir, or 100 μm ganciclovir examined with CMV‐*UL83* qPCR after 3 days (*n* = 4). Data are shown as fold change compared to control and examined with paired t‐tests. (J) Immunoblot of LNCaP‐abl 4 days after transfection with control RNAi, IE1/2 RNAi (1). CMV‐US28, CMV‐UL97, and β‐actin expression was examined, quantified (*n* = 3) and analyzed with one‐sample t‐test. kDa is kilodalton. UL97 isoforms are labeled with asterisks. (K) CMV DNA abundance determined with CMV‐*UL83* qPCR in DU145 4 days after transfection with IE1/2 RNAi (1) compared to control RNAi (*n* = 6). Data are displayed as negative fold change. Box plots in (B) and (E) show median; boxes 25–75 percentiles and error bars are max and min. *P < 0.05, **P < 0.01, ***P < 0.0001, ns, nonsignificant. Nuclei are labeled in blue with DAPI in (B), (E) and (G). CMV, Human cytomegalovirus; CRPC, Castration‐resistant prostate cancer; CSPC, Castration‐sensitive prostate cancer; FISH = Fluorescence *in situ* hybridization; IHC, Immunohistochemistry; IE1/2, Immediate early 1 and 2; RNAi, RNA interference.

To examine to which extent prostate cancer cells are infected with CMV, we analyzed 20 tumors in prostatectomy specimens from patients with prostate cancer (Fig. 2A; Fig. S2A), two incidental tumors found postmortem in subjects without previously known prostate pathology and bone metastases from 10 patients in a separate cohort (Fig. 2A). CMV was detected by CMV pp71 IHC in 17/20 (85%) of primary tumors (Fig. 2A), with 10 having very high abundance of CMV (90–100% of cancer cells infected, Fig. 2B). In prostate samples from the three patients with CMV negative primary tumors, CMV was also absent from the benign epithelium (Fig. S2B ). In prostatectomy samples with CMV‐positive tumors, the surrounding benign epithelium was also CMV infected (Fig. S2B ). Of two incidental prostate cancers in two postmortem donors, one tumor was CMV^+^ and the other tumor was CMV^−^ (Fig. 2A; Fig. S2C ).

Primary prostate cancer cells often metastasize to distal sites such as bone. CMV was detected by CMV‐pp71 IHC in bone metastases in 7/10 (70%) of patients (Fig. 2A,C,D, Fig. S3A). In addition, four examined bone metastases were positive for CMV‐*UL37* qPCR. In five patients, two to three metastases were examined, with 9/13 (69%) CMV^+^ metastases examined being homogenously infected whereas four metastases were heterogeneously infected (Fig. 2D; Fig. S3A). These results show that CMV infection is common in primary and metastasized prostate cancer. We have recently shown that CMV abundance or seropositivity is not associated with Gleason score, T‐stage, or advanced prostate cancer stage at diagnosis, but CMV seropositive prostate cancer patients have increased risk of prostate cancer mortality and have high abundance of CMV in their tumors [6]. Taken together, the presence of CMV in a large fraction of prostate cancers and its association with poor prognosis suggests that CMV may have tumor‐promoting functions and can be an actionable therapeutic target.

Nearly all primary prostate cancers are adenocarcinomas with AR expression and active androgen signaling, a core pathway in prostate cancer that promotes growth. As advanced prostate cancer is treated with anti‐androgen therapy, they ultimately develop from castration‐sensitive prostate cancer (CSPC) to lethal castration‐resistant prostate cancer (CRPC). Most often, reactivated androgen signaling drives CRPC, but it can also arise through non‐AR‐driven mechanisms, a disease state characterized by worse outcome and fewer treatment options [1, 2]. Phenotypes of bone metastases from 10 patients were characterized by transcriptome analyses [2]. Six patients had metastases with high AR activity (MetA) and four patients were of a dedifferentiated phenotype (MetB) [2]. CMV infection was present in bone metastases from 5/6 patients with MetA and 2/4 patients with MetB (Fig. 2D). Importantly, this concludes that CMV can be present in advanced prostate cancer irrespective of castration resistance and phenotype, making its biological and therapeutic relevance in metastatic prostate cancer intriguing to explore.

### 3.3 CMV in prostate cancer cell lines and mechanism of virus maintenance

To determine if CMV influences cancer‐promoting features such as cell viability, cell proliferation or AR signaling, we turned to prostate cancer models. Infecting prostate cancer cells with purified CMV virions may not accurately mimic latent *in vivo* prostate cancer infection. We therefore screened whether well‐established cell lines derived from patients with advanced prostate cancer were endogenously CMV infected, thereby offering a natural experimental system. We analyzed seven commonly studied prostate cancer cell lines, representing CSPC (LNCaP, LAPC‐4, VCaP), AR‐driven/adenocarcinoma CRPC (LNCaP‐abl, LREX’), and non‐AR‐driven CPRC (DU145, PC3). In implanted DU145 prostate cancer cells in mice as xenografts, CMV DNA was detected in most cancer cells (pan‐keratin^+^) by *in situ* hybridization and human CMV was, as expected, absent in mouse cells (pan‐keratin^−^, Fig. 2E). Notably, all seven analyzed cell lines carried CMV, as indicated by being CMV‐*UL83* DNA qPCR^+^ (Fig. 2F), and the mouse prostate cancer cell line MyC‐CaP was CMV‐*UL83* qPCR negative (Figs 1H, 2F). Cell lines were also CMV UL97 and US28 protein positive (Fig. S4A).

RNA interference (RNAi) against the CMV genes *UL97*, *US28*, and *UL122‐UL123* reduced expression of the corresponding proteins, validating their presence in prostate cancer cell lines (Fig. 2G, Fig. S4B–E), although we were unable to detect CMV RNA by RT‐qPCR (Fig. S4F S). To test for functional evidence of CMV in prostate cancer cells, we asked if UL97 function as a kinase that phosphorylates the Retinoblastoma protein (Rb) [53] was conserved. Notably, both *UL97* RNAi and treatment with the UL97 kinase inhibitor maribavir resulted in reduced phosphorylation of Rb on Ser^807^/Ser^811^ in prostate cancer cells (Fig. 2H; Fig. S4G). We conclude that CMV infection is very common in prostate cancer cell lines, irrespective of their phenotype, with detectable viral DNA, proteins, and CMV enzymatic activity.

We next asked how CMV is maintained in prostate cancer cells. Absence of detectable RNA indicated a latent viral state. Active/lytic infection is inhibited by current antiviral drugs whereas latent infection remains. Treatment with ganciclovir, which blocks viral DNA synthesis, and maribavir, which also inhibits virion production, did not reduce levels of CMV DNA examined by qPCR in prostate cancer cells (Fig. 2I). This finding suggests that CMV is not maintained through virion production and reinfection and infers that the infection is latent.

We asked how latent CMV is maintained in prostate cancer cells and if latent CMV could be abolished. The well‐characterized CMV gene locus *UL122‐UL123* encoding the proteins immediate early 1 and 2 (IE1/2) regulates the viral life cycle [54] and is necessary for latent CMV genome replication in experimental models of CMV infection in hematopoietic lineage cells [25]. We asked if inhibition of IE1/2 in CMV^+^ prostate cancer cells could impair latent infection. RNAi against *UL122‐UL123* (IE1/2 RNAi) reduced the levels of IE1/2 protein (Fig. 2G; Fig. S4B) as well as of other CMV proteins (Fig. 2J; Fig. S4H). Furthermore, the presence of CMV DNA was decreased more than fivefold by IE1/2 RNAi (Fig. 2K), establishing that *UL122‐UL123* is required to maintain CMV infection in prostate cancer cell lines. In summary, these findings expand upon known functions of *UL122‐UL123* and suggest that latent CMV can be removed from infected cells by targeting this pathway, offering an experimental system to study the functional role of CMV in prostate cancer cells.

### 3.4 CMV promotes prostate cancer cell survival, proliferation, and androgen receptor signaling

**Fig. 3:**
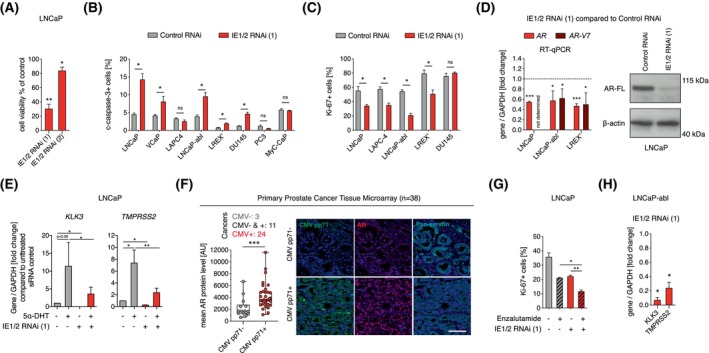
Endogenous CMV promotes cell viability and androgen receptor expression in prostate cancer. (A) Cell viability, determined with the CellTiter‐Glo 2.0 Cell Viability Assay, in LNCaP 4 days after transfection with IE1/2 RNAi (1) (*n* = 6) or IE1/2 RNAi (2) (*n* = 4) compared to control RNAi. (B) Cells transfected with control RNAi or IE1/2 RNAi (1) analyzed after 3 or 4 days for % cleaved caspase‐3+ cells (*n* = 3, except for VCaP and LNCaP‐abl: *n* = 4). MyC‐CaP is a mouse prostate cancer cell line. (C) Cells transfected with control RNAi or IE1/2 RNAi (1) and analyzed after 3 or 4 days for percentage of Ki‐67^+^ cells (*n* = 3). (D) Cells transfected with control RNAi or IE1/2 RNAi (1) was examined for *AR* and *AR‐V7* gene expression with RT‐qPCR (LNCaP, *n* = 3; LNCaP‐abl and LREX’, *n* = 4) and full‐length AR (AR‐FL) protein expression with western blot 3 days after transfection. (E) LNCaP grown in charcoal‐stripped FBS and transfected with control RNAi or IE1/2 RNAi (1) 4 days (*n* = 3). Cells were treated with 10 nm 5α‐DHT one day after which gene expression of the AR target genes *TMPRSS2* and *KLK3* was examined. Gray bars are cells treated control RNAi, and red bars are cells treated with IE1/2 RNAi (1). (F) An association between CMV pp71 presence in cancer cells and AR protein levels in nuclei (AU; arbitrary unit) was examined in a tissue microarray with primary prostate cancer (*n* = 38). Pan‐keratin was used as a marker of epithelial cells. Each dot in graph is mean AR protein level of all CMV pp71‐ cells (gray) in one tumor and CMV pp71+ cells (red) in one tumor, compared with Mann–Whitney test. Three samples contained no CMV+ tumor cells, 11 samples contained both CMV+ and CMV− tumor cells and 24 samples contained only CMV+ tumor cells. Box plot shows median; box shows 25–75 percentiles and error bars show max and min. Scale bar: 100 μm. (G) Percentage of Ki‐67+ cells in LNCaP transfected with control RNAi or IE1/2 RNAi (1) and treated with vehicle or 10 μm enzalutamide the following day (*n* = 3). (H) Gene expression analysis in IE1/2 RNAi (1) treated LNCaP‐abl compared to control RNAi 4 days after transfection (*n* = 3). Cell nuclei in (F) are labeled in blue with DAPI. Data in bar graphs are shown as mean. Error bars in bar graphs are SEM. RT‐qPCR results are shown as fold change of control. Unless otherwise stated, treatment effects were examined with paired *t*‐tests. *P < 0.05, **P < 0.01, ***P < 0.001, ns, nonsignificant. 5α‐DHT, 5α‐dihydrotestosterone; AR, Androgen receptor; CMV, human cytomegalovirus; IE1/2, immediate early 1 and 2; RNAi, RNA interference.

As we had established the presence of CMV and a loss‐of‐function experimental setup *in vitro* by IE1/2 knockdown, we were now able to ask how CMV influences cellular functions in prostate cancer cell lines. We used IE1/2 RNAi to assess the effect of CMV inhibition in seven CMV^+^ prostate cancer cell lines representing CSPC (LNCaP, LAPC‐4, VCaP), AR‐driven/adenocarcinoma CRPC (LNCaP‐abl, LREX’), and non‐AR‐driven CRPC (DU145, PC3). IE1/2 RNAi mediated reduction of CMV reduced viability (determined with a CellTiter‐Glo assay) of prostate cancer cells (Fig. 3A; Fig. S4I) and increased levels of the apoptosis marker cleaved caspase‐3 in 5/7 (71%) of the examined prostate cancer cell lines, independent of phenotype (Fig. 3B). As expected, apoptosis was not induced by IE1/2 RNAi in the CMV^−^ mouse prostate cancer cell line MyC‐CaP (Fig. 3B) nor did it alter cell viability in the human fibroblast cell line WI‐38 (Fig. S4J). We conclude that CMV inhibition reduced cell survival in models of CSPC and CRPC independent of AR expression.

Next, we asked if CMV promoted prostate cancer cell proliferation. IE1/2 RNAi reduced the proportion of cells being positive for the cell proliferation marker Ki‐67 in AR‐expressing CSPC and CRPC cell lines, but not in non‐AR‐driven CRPC (Fig. 3C). To assess whether CMV can influence androgen signaling, a key pathway driving prostate cancer progression, we transfected CSPC cells with IE1/2 RNAi. This resulted in reduced *AR* gene expression and AR protein levels (Fig. 3D), as well as reduced expression of the androgen‐regulated genes *KLK3* and *TMPRSS2* in response to 5α‐dihydrotestosterone (5α‐DHT) (Fig. 3E). Moreover, we found that CMV‐infected cells (CMV‐pp71^+^) expressed higher levels of AR protein than uninfected (CMV pp71^−^) cells in primary prostate cancer samples (*n* = 38) (Fig. 3F), suggesting that CMV increases AR levels in prostate cancer cells also *in vivo*. In summary, these findings show that CMV promotes proliferation and androgen signaling in AR‐expressing prostate cancer cells.

Anti‐androgen therapy is the foundation against advanced prostate cancer. We asked if CMV inhibition could have additive effects to anti‐androgen therapy in CSPC and CRPC. In the CSPC cell line LNCaP, combining IE1/2 RNAi and the AR inhibitor enzalutamide further reduced proliferation compared to either treatment alone (Fig. 3G). Increased activity of AR and the AR splice variant AR‐V7 by higher expression or gain of function mutations is a hallmark of CRPC and may act to confer resistance to anti‐androgen therapy [55]. These processes are mimicked in the cell line LNCaP‐abl and LREX’. IE1/2 RNAi decreased protein levels and expression of *AR* and *AR‐V7* and their target genes *KLK3* and *TMPRSS2* in models of CRPC (Fig. 3D,H). Taken together, these findings demonstrate that CMV inhibition can attenuate androgen signaling in CRPC and imply that CMV inhibition may have additive effects to androgen receptor‐targeting pharmaceutical compounds in CMV^+^ prostate cancer.

### 3.5 The effect of aciclovir in prostate cancer models and in a large population cohort

**Fig. 4:**
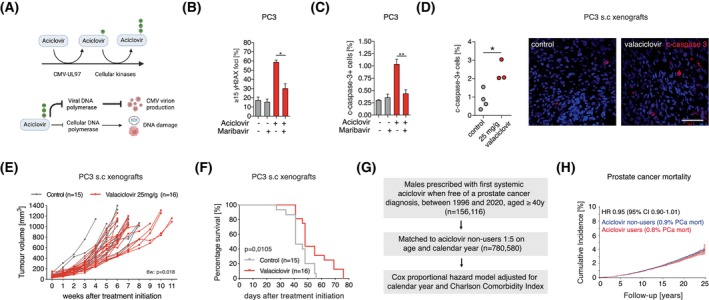
Aciclovir in prostate cancer. (A) Illustration, made with Biorender.com, of aciclovir activation and mechanism of action of activated, phosphorylated (P) aciclovir. (B, C) In PC3, maribavir treatment (10 μm) reduced the ability of aciclovir to promote yH2AX foci (*n* = 3) (B) and to induce apoptosis (*n* = 3) (C), as analyzed with paired t‐tests. Error bars are shown as SEM. (D) Mice were untreated (control, *n* = 4) or treated with valaciclovir (*n* = 3) 17–21 days. Aciclovir‐induced apoptosis (cleaved caspase‐3^+^ cells) in PC3 xenografts. Percentage of cleaved caspase‐3^+^ cells was compared with unpaired *t*‐test. Scale bar: 25 μm. (E) PC3 xenograft volume over time (weeks after treatment initiation) in control (*n* = 15) and valaciclovir‐treated animals (*n* = 16). Repeated measurement mixed‐effect analysis was used to compare tumor volume over time and multiple comparison statistics were assessed at 6 weeks. (F) Kaplan–Meier survival curve comparing survival of control (*n* = 15) and valaciclovir (*n* = 16) treated animals. Survival was compared using log‐rank test. (G) Flow chart of study design of aciclovir population cohort and prostate cancer mortality. (H) Cumulative incidence (%) of prostate cancer mortality in aciclovir nonusers (blue line) and aciclovir users (red line). Shaded blue and red areas represent 95% confidence intervals (CI). HR, hazard ratio. *P < 0.05, **P < 0.01.

Aciclovir and ganciclovir are common and well‐tolerated nucleotide analogue anti‐herpes virus drugs, that are both activated through its phosphorylation by CMV UL97 kinase [56, 57]. Aciclovir is clinically used as CMV prophylactic treatment in transplant patients [58]. A case study reported that two prostate cancer patients with shingles (caused by the herpes virus varicella zoster) treated for 2 weeks with valaciclovir, an aciclovir pro‐drug, obtained a marked and lasting drop in serum prostate‐specific antigen [59]. Although several explanations for aciclovir associated effects on serum prostate‐specific antigen are plausible, we hypothesized that CMV may be its target and be used to treat prostate cancer. In addition to inhibiting viral DNA synthesis, activated aciclovir can inhibit human DNA polymerase with low affinity, introducing DNA damage and cell death [60] in the absence of an actively replicating virus (Fig. 4A). Similarly, activated ganciclovir can induce cell death *in vivo* [61].

Anticancer effects of aciclovir were tested in prostate cancer cell lines. Aciclovir increased the number of γH2AX foci per nuclei (Fig. 4B; Fig. S5A), a marker of double‐stranded DNA damage, and induced apoptosis, as determined by the percentage of cleaved caspase‐3^+^ cells, in six prostate cancer cell lines (Fig. 4C; Fig. S5B). The related drug ganciclovir resulted in a similar response (Fig. S5C,D). This response was abolished when CMV‐UL97 was inhibited with maribavir, down‐regulated with UL97 RNAi (Fig. 4B,C; Fig. S5C,E,F) or upon IE1/2 RNAi (Fig. S5G ), demonstrating CMV‐dependent effects of aciclovir and ganciclovir in latent CMV‐infected prostate cancer cells. Further studies focused on aciclovir as it showed similar effects to ganciclovir in prostate cancer cells and has a milder side effect profile compared to aciclovir. Treatment with aciclovir for 5 days followed by 7 days of no treatment enhanced its apoptosis inducing effect (Fig. S5H,I) which was accompanied by an increase in cell proliferation (Fig. S5J) and DNA damage (Fig. S5K).

To assess *in vivo* effects of aciclovir, mice bearing PC3 xenografts were administered valaciclovir orally (25 mg·g^‐1^ chow), which resulted in aciclovir serum concentrations comparable to therapeutic concentrations in humans (Fig. S5L) [62, 63]. Valaciclovir‐induced selective apoptosis in cancer cells (Fig. 4D; Fig. S5M) and tumor growth was reduced by 16%, comparing mean volume of tumors at 6 weeks (Fig. 4E; *P* = 0.018). Valaciclovir (*n* = 16) increased survival (Fig. 4F; log‐rank test; *P* = 0.011) compared to control mice (*n* = 15), with 31% (5/16) of animals alive 60 days after treatment initiation compared to 0% (0/15) of control (Fisher's exact test *P* = 0.043). In summary, aciclovir‐induced apoptosis in CMV^+^ prostate cancer cell lines and resulted in a slight reduction of tumor growth and increased survival in a xenotransplantation model of prostate cancer.

To get a first indication of the clinical potential of aciclovir to treat prostate cancer, we conducted a large cohort study evaluating if men prescribed aciclovir had decreased risk of prostate cancer mortality using Danish population‐based linked registries [38, 39, 40, 41, 64]. Since prostate cancer is common, slow growing and may be present years before diagnosis, we examined risk of prostate cancer mortality in all men aged 40 and older (Fig. 4G). Systemic aciclovir/valaciclovir users (from here referred to as aciclovir users; *n* = 156 116) were matched by age at first prescription, defined as start of follow‐up, and calendar year to nonusers (*n* = 780 580; Fig. 4G; Table S1). All aciclovir users, independent of the number of aciclovir prescriptions, were included. In Cox proportional hazard models adjusted for age, calendar year, and comorbidity index (Table S1), aciclovir users had a lower, but not statistically significant, prostate cancer mortality, adjusted HR 0.95 (95% CI 0.90–1.01, *P* = 0.09) (Fig. 4H).

### 3.6 Anti‐CMV drugs can be repurposed against prostate cancer

The limited prostate cancer response to aciclovir prompted us to investigate whether other drugs against CMV could be more efficient. We studied both chemotherapeutic drugs and novel antiviral compounds. The chemotherapeutic drugs ellipticine and mithramycin A, which both are DNA intercalators, reduce latent CMV genome replication in experimental models [25]. Mithramycin A is sometimes used to treat patients with testicular cancer [65] and cancer‐related hypercalcemia [66], whereas ellipticine is not in clinical use, but derivatives more suitable for human use are being developed. Viral proteins are often multifunctional and operative both during latency and active replication. There is therefore a rational for evaluating drugs targeting CMV proteins in prostate cancer cells and examine if these could be repurposed. We examined the novel anti‐CMV drugs maribavir, a CMV UL97 kinase inhibitor, and letermovir, a CMV UL56 inhibitor. Both drugs are well‐tolerated and in clinical use [67, 68].

**Fig. 5:**
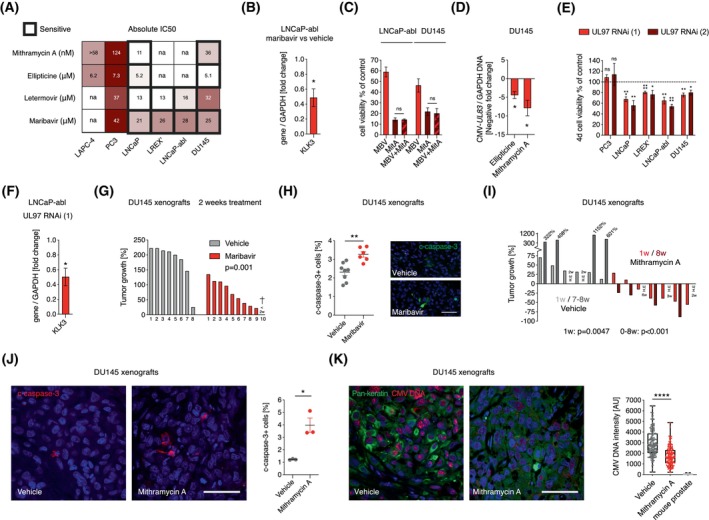
CMV can be therapeutically targeted in prostate cancer with chemotherapy and novel antiviral drugs. (A) Heat map with absolute IC50 in cell lines treated with mithramycin A 3 days (LNCaP, LAPC‐4, *n* = 4; PC3, DU145, *n* = 5 nm), ellipticine 3 days (*n* = 3 except LNCaP, *n* = 4 μm), letermovir 6 days (*n* = 3, μm), or maribavir 6 days (*n* = 3 all cell lines except LNCaP‐abl, *n* = 4 μm). Thick boxes show the most drug‐sensitive cell lines. Lighter colors represent lower absolute IC50 and therefore higher sensitivity. Na = not analyzed. (B) Gene expression in LNCaP‐abl after 4 days of maribavir treatment compared to vehicle (*n* = 4). Data are displayed as fold change. (C) LNCaP‐abl and DU145 treated with 30 μm maribavir (MBV), 30 nm mithramycin A (MitA) or both. Cell viability was examined after 3 days of treatment (*n* = 3). (D) CMV DNA abundance determined with CMV‐*UL83* qPCR in DU145 4 days after treatment with 3 μm ellipticine or 30 nm mithramycin A compared to vehicle (*n* = 3). Data are displayed as negative fold change. (E) Cell viability, determined with the CellTiter‐Glo 2.0 Cell Viability Assay, evaluated by one‐sample *t*‐tests 4 days after transfection with control RNAi, UL97 RNAi (1) (PC3, *n* = 4; LNCaP, *n* = 6; LREX’, *n* = 8; LNCaP‐abl, *n* = 6, DU145 *n* = 5) or UL97 RNAi (2) (PC3, *n* = 4; LNCaP, *n* = 5; LREX’, *n* = 6; LNCaP‐abl, *n* = 6, DU145 *n* = 4). (F) *KLK3* expression examined after 4‐day treatment with UL97 RNAi (1) compared to control RNAi in LNCaP‐abl (*n* = 5). (G) DU145 tumor growth in percentage after 2 weeks treatment with vehicle or 100 mg·kg^−1^ maribavir two times per day. (H) Percentage of cleaved caspase‐3^+^ cells in vehicle (*n* = 8) and maribavir (*n* = 6) treated DU145 xenografts. Scale bar: 50 μm. Cell nuclei are labeled in blue with DAPI. (I) Alteration of tumor size in percentage 1 week (w) and 7–8 weeks after vehicle (*n* = 6) or mithramycin A treatment (*n* = 7). 1 week: Mann–Whitney *t*‐test; 0–8 weeks: Repeated measures (RM) mixed‐model. HE = Humane endpoint (tumor ulcers: *n* = 2 control, *n* = 1 mithramycin A; found dead *n* = 1 mithramycin A; unwell *n* = 1 mithramycin A). (J) Percentage of cleaved caspase‐3^+^ cells in DU145 xenografts in vehicle and mithramycin A treated animals (*n* = 3 per treatment, all datapoints are shown in graph). Scale bar: 50 μm. Cell nuclei are labeled in blue with DAPI. (K) CMV DNA examined by CMV FISH in tumors and mouse prostate after 7–8 weeks of treatment. Cell nuclei are labeled in blue with DAPI. Pan‐keratin was used as a marker for epithelial cells. CMV DNA in pan‐keratin+ cells, measured by nuclear intensity, was compared between vehicle (*n* = 3) and mithramycin A (*n* = 3) treated mice. Epithelial cells visualized with DAPI were quantified in mouse prostate. Scale bar: 50 μm. Box plot show median; box is 25–75 percentiles and error bars show max and min. RT‐qPCR results are shown as fold change of control. Unpaired Student's t‐test in (G–H) and (J–K). Paired *t*‐tests were performed in (B–F). Bar graphs are shown with mean value and error bars are SEM. *P < 0.05, **P < 0.01, ***P < 0.001, ****P < 0.0001, ns, nonsignificant. CMV, Human cytomegalovirus; FISH, Fluorescence *in situ* hybridization; RNAi, RNA interference.

Prostate cancer cell lines responding to IE1/2 RNAi with apoptosis were also most sensitive to mithramycin A, ellipticine, and maribavir, determined with a cell viability assay (Fig. 5A; Fig. S6A). These drugs drastically reduced cell viability after 3–6 days (Fig. 5A), being both faster and more efficient than aciclovir (Fig. S6B). The drugs induced apoptosis and reduced proliferation in cell lines representing both CSPC and CRPC (Fig. S6C, D, E, F) and reduced AR target gene expression [69] (Fig. 5B). Compared to maribavir, the effect of letermovir was limited to cell lines expressing AR (Fig. 5A). There were no additive effects of IE1/2 RNAi and ellipticine or mithramycin A on apoptosis or proliferation (Fig. S6G,H), nor did maribavir and mithramycin A have additive effects on cell viability (Fig. 5C), suggesting that they all act through the same CMV‐dependent mechanism.

We asked how mithramycin A, ellipticine, and maribavir exerted their effects through CMV inhibition. Mithramycin A and ellipticine reduced levels of CMV DNA in prostate cancer cells (Fig. 5D), in line with their known negative effects on latent genome maintenance [25]. But whereas mithramycin A and ellipticine target proteins SP1 and TOPIIB are responsible for latent genome replication in hematopoietic cells, knockdown of these proteins in prostate cancer cells did not mimic mithramycin A or ellipticine induced phenotypes (Fig. S7A–E). Mithramycin A and ellipticine are both DNA intercalators. To examine if other chemotherapeutic drugs with DNA interacting properties are also CMV inhibitors, we treated prostate cancer cells with cisplatin or etoposide. These drugs only readily reduced cell viability in one out of four cell lines (Fig. S7F) and etoposide did not reduce CMV DNA abundance (Fig. S7G), suggesting some specificity to mithramycin A and ellipticine.

The CMV UL97 kinase has not been reported as a CMV latency regulator, and maribavir did not reduce CMV abundance in prostate cancer cells (Fig. 2I), supporting a nonessential role for UL97 in maintaining latency. However, this finding suggests that maribavir targets a key prostate cancer driver as maribavir treatment mimics several features of IE1/2 RNAi and mithramycin A or ellipticine treatment. Similar to maribavir, inhibiting its target protein UL97 with RNAi reduced cell viability (Fig. 5E) and AR target gene expression (Fig. 5F). These findings imply that maribavir specifically targets CMV in prostate cancer cells.

We asked whether mithramycin A or maribavir may affect prostate cancer cells in a xenograft model in mice. Mice with established DU145 xenografts were given 100 mg·kg^−1^ maribavir (*n* = 10) or vehicle (*n* = 8) twice daily every weekday. Although animals lost weight the first week of maribavir treatment, they then stabilized and kept a steady weight throughout the remaining treatment period (Fig. S8A), but continuous treatment for more than 3.5 weeks was not well tolerated. In line with the *in vitro* results (Fig. S6F), maribavir did not change the number of proliferating cells in DU145 xenografts (Fig. S8B). Tumor growth was reduced in mice treated with maribavir examined after 2 weeks (Fig. 5G). Maribavir‐induced reduction of tumor growth was accompanied by increased apoptotic activity in the tumors (Fig. 5H). Mithramycin A induced shrinkage of DU145 cell xenografts after 7 days of treatment that was durable over an 8‐week period (Fig. 5I), displayed signs of active apoptosis (Fig. 5J) [70], and reduced CMV genome abundance (Fig. 5K). To summarize, these results show that CMV targeted drugs such as mithramycin A and maribavir have potential clinical benefit against prostate cancer.

## 4 Discussion

Here we report that CMV infection is very common in the healthy and malignant prostate epithelium. Out of seven commonly studied prostate cancer cell lines, all were CMV infected. All evidence point to a persistent latent viral state in prostate cells in which viral proteins are expressed and functional but virions are not produced, in line with current paradigms on CMV latency in other cell types. CMV abundance could be drastically reduced by CMV‐IE1/2 RNAi and by several pharmaceutical compounds, which enabled us to study the functional role of CMV in prostate cancer models. CMV promoted proliferation, cell survival, and androgen signaling in castration‐sensitive and resistant prostate cancer cell lines. CMV abundance in prostate epithelia increased with age, and CMV^+^ prostate cancer cells had higher levels of AR protein compared to uninfected cancer cells, findings that suggest that the effects of CMV we identified *in vitro* are operational also in humans *in vivo*. Aciclovir showed modest effects on tumor growth in a preclinical model, and aciclovir usage in clinical practice was not associated with significantly reduced prostate cancer mortality at the dose and duration studied. Old chemotherapeutic drugs such as mithramycin A and novel CMV drugs including the CMV‐UL97 inhibitor maribavir had more marked effects on cell viability in CMV‐dependent prostate cancer cells. These results prompt investigation into the clinical use of anti‐CMV drugs such as mithramycin A and maribavir for the treatment of prostate cancer.

The presence, characteristics, and role of CMV in tissues and in diseases such as cancer are challenging to study and have hampered progress [31], not least due to inconsistencies in viral detection techniques. It is broadly accepted that cells of the hematopoietic lineage are infected by CMV, although CMV RNA is difficult to detect in chronic CMV infection [24, 26, 29, 49]. Viral gene expression during latency can also be undetectable in cells of human papilloma virus‐infected tumors [71], suggesting that general mechanisms of gene expression regulation may exist for several DNA viruses. The presence and cellular localization of viral proteins has rarely been assessed in endogenously CMV‐infected cells during latency, and knowledge on CMV proteins therefore highly relies on *in vitro* experiments. CMV IE proteins have classically been viewed as nuclear proteins during active replication with no expression during latency. However, current paradigms state that a diverse array of CMV genes, including IE genes, can indeed be expressed during latency [26], albeit at lower levels. Viral proteins can have different functions depending on viral and cellular state, and specific cellular localizations can also differ, as exemplified with CMV pp71 [46]. In this study, IE proteins were not exclusively expressed in cell nuclei, a phenotype that has also been described in other cancer types. These findings warrant further exploration of CMV protein expression in latency, such as cells of the hematopoietic lineage, and cell compartment‐specific protein functions. Coherent detection of CMV with several antibodies and with CMV DNA, strengthen our conclusions of a common CMV presence in prostate epithelium and also imply that detection bias due to variations in CMV strains or mutations was negligible.

Most commonly, patches of epithelial cells, and not single cells, were CMV positive, suggestive of cellular clones or hotspots for CMV reinfection. If CMV were to actively replicate and commonly infect neighboring cells, lysis of cells and an inflammatory response would be expected. The lack of these findings [6] implies that CMV persists in epithelial cells via other mechanisms, such as replication of latent viral genomes and passaging of these to daughter cells. The notion that CMV may not actively replicate in transformed cells [72] speaks for this paradigm. Our cohort of postmortem donors allowed us to thoroughly address CMV presence with multiple methods, but could potentially introduce detection bias. Varicella zoster virus may reactivate postmortem [73], but it is unclear if this phenomenon occurs with CMV. Absence of a correlation between postmortem interval and CMV protein levels in prostates and low detection of CMV DNA postmortem [74] suggest that it might not reactivate in these circumstances.

The majority of analyzed prostate cancer cell lines were dependent on endogenous CMV for cell survival, reminiscent of addiction to oncogenes such as MYC, a protein that, similar to latent viruses, promotes immune evasion [75]. CMV, with around 700 putative open reading frames [76], can alter many cellular processes, including those that promote cell survival and proliferation, for viral replication and dissemination to proceed [11]. In experimental models, CMV can promote tumorigenesis [77] and promote tumor progression of established cancer [78], but the generalizability of these findings is unclear, since the cellular consequences of CMV protein expression and functions may differ in a chronic or latent viral state. It is therefore important to study viral effects on cancer in its proper context in its natural infectious states, as in this study. CMV has, among other malignancies, been proposed to infect glioblastoma, and perturbation of IE1/2 in endogenously CMV‐infected glioblastoma stem cells reduces cell viability [44], perhaps pointing to shared functions of CMV in several tumor types. In our study, knockdown of CMV‐IE1/2 in prostate cancer cells resulted in attenuation of CMV DNA, and it is therefore not certain whether IE1/2 proteins themselves act to preserve cell viability. The CMV attenuation phenotype was only partly mimicked by UL97 inhibition, suggesting that more CMV genes are at play.

Cell lines are by design passaged multiple times and may differ from their original phenotypes *in vivo*, which could also be the case for CMV, causing potential limitations in the generalizability of our findings. Other *ex vivo* models more closely resembling *in vivo* properties, such as patient‐derived xenografts and organoids, are attractive models to further explore.

Merkel cell polyomavirus is an oncogenic virus, and Merkel cell carcinoma cells are addicted to the expression of a viral oncogene *in vitro* [79]. Although prostate cancer cells may require CMV for survival, one may not label CMV an oncogenic virus. For instance, CMV was not essential for prostate tumorigenesis as CMV^−^ tumors and partly CMV^−^ tumors can arise. Furthermore, there is no link between CMV seropositivity and prostate cancer risk [6]. In addition, cancers caused by viruses are often prone to arise upon immunosuppression, but no such association has been found for prostate cancer [80]. Rather, one could label CMV as an onco‐modulating virus, making CMV an attractive drug target for established cancers, even in its advanced stages.

In addition to cell survival, CMV promoted proliferation. CMV influences AR signaling, a known pro‐proliferation pathway in prostate cancer by promoting gene expression of the *AR*. UL97 inhibition reduced AR target gene expression, but other CMV genes may also be AR regulators. Much is known about cofactors and upstream regulators driving AR signaling, but further studies are required to elucidate mechanisms for CMV‐driven AR signaling. Another potential pro‐proliferative mechanism of CMV could be through phosphorylation and inactivation of Rb by UL97 (Fig. 2E) [53], as active Rb generally binds to E2F resulting in cell cycle inhibition. The percentage of cells in the cell cycle was not altered by CMV inhibition in Rb^low^ DU145 cells, supporting this hypothesis. But Rb regulation and function in prostate cancer are intricate and loss of function is more common when the disease is advanced, unlikely to be solely attributed to CMV.

Importantly, several methods of CMV inhibition reduced cell viability in models of CRPC, a disease state with limited treatment options. A multitude of resistance mechanisms have been described, including AR mutations, *AR* or *AR‐V7* upregulation, and lineage switching from adenocarcinoma to androgen indifferent basal/neuroendocrine cell states [55]. CMV was present in several phenotypes of advanced prostate cancer *in vitro* and *in vivo*, which implies that CMV is not sufficient to drive cancer cell lineage commitment. In CRPC with high AR activity, CMV inhibition lowered expression of *AR* and *AR‐V7*, a function that can be therapeutically exploited. As upregulation of *AR* and *AR‐V7* is associated with the development of treatment resistance, these data pose the question if CMV may potentially exacerbate antiandrogen resistance.

Although aciclovir induced CMV‐dependent DNA damage and apoptosis *in vitro*, the *in vivo* effect was modest in a xenograft model, and we found no association between systemic (val)aciclovir usage and prostate cancer mortality. It is not possible to infer causality from our observational study, and the study design used is prone to bias such as confounding by indication. Although our results speak against the clinical activity of aciclovir, we speculate that a potential aciclovir effect may be most evident in CMV seropositive men. Identifying possible super responders, exploring the timing and duration of treatment, dosing schemes, and combination therapies will be important to further evaluate possible clinical benefits.

The novel antiviral drugs letermovir and maribavir had more substantial effects on prostate cancer cell viability than aciclovir at therapeutic doses, with maribavir having the broadest effects. Mithramycin A most closely mimicked experimental CMV loss, but systemic toxicities hamper general clinical use. Milder mithramycin A analogues and novel drug delivery systems could potentially be used to overcome this obstacle. Several other viruses that infect and promote cancers have not been readily possible to target with antiviral therapies, due to their latent nature. Here we show that it is feasible to target an endogenous DNA virus as cancer therapy. That CMV seropositivity in prostate cancer patients is associated with increased mortality [6] and our demonstration here that CMV promotes core prostate cancer pathways and that its inhibition reduces tumor growth, motivates studies of the potential benefit of anti‐CMV therapies to extend the lives of prostate cancer patients.

## 5 Conclusions

We conclude that CMV infection is common in prostate cancer and has the potential to be widespread. CMV inhibition reduces prostate cancer cell viability, and several potential treatment strategies by targeting CMV are proposed.

## Conflict of interest

The authors declare no potential conflicts of interest.

## Author contributions

JF and JC conceived the study. JF supervised the study. JC performed cell experiments, analysis of human tissue and blood. JC, MS, MZ, C‐JE, and AS performed and analyzed animal experiments. KA and HD procured tissue and blood from men postmortem. KA collected postmortem tissues and blood and stained FFPE sections with H&E. HD performed histological assessments of prostate tissue and reviewed medical history. AT and AS collected data on human samples. PW designed the collection of bone metastases and provided tissues for analysis. AB designed the collection of bone metastases and performed tissue analyses. LP, H‐OA, and HTS designed epidemiological studies. LP performed statistical analysis of epidemiological studies. JF and JC wrote the manuscript with intellectual input from all authors.

## Peer review

The peer review history for this article is available at https://www.webofscience.com/api/gateway/wos/peer‐review/10.1002/1878‐0261.70073.

## Supporting information


**Fig. S1.** CMV DNA and protein in the prostate.
**Fig. S2.** CMV in prostate cancer.
**Fig. S3.** CMV in prostate cancer metastases.
**Fig. S4.** CMV proteins are detected in prostate cancer cells and can be reduced with RNAi.
**Fig. S5.** Pre‐clinical evaluation of aciclovir.
**Fig. S6.** Therapeutic targeting of CMV in prostate cancer.
**Fig. S7.** Mechanism of ellipticine and mithramycin A.
**Fig. S8.** Maribavir in a prostate cancer xenograft model.
**Table S1.** Characteristics of aciclovir epidemiology cohort.
**Table S2.** Small interfering RNA sequences.
**Table S3.** List of custom primer/probes used for qPCR.
**Table S4.** List of custom primer/probes used for RT‐qPCR.

## Data Availability

All data are available in the main text or the supplementary materials.
