# Supplementary material for: Cytomegalovirus infection is common in prostate cancer and antiviral therapies inhibit progression in disease models
Source: Mol Oncol. 2025 Jun 10;19(11):3035–59. doi: 10.1002/1878-0261.70073 (PMC12591316; doi:10.1002/1878-0261.70073)
Supplement: Supplementary file 1 — Fig. S1. CMV DNA and protein in the prostate. Fig. S2. CMV in prostate cancer. Fig. S3. CMV in prostate cancer metastases. Fig. S4. CMV proteins are detected in prostate cancer cells and can be reduced with RNAi. Fig. S5. Pre‐clinical evaluation of aciclovir. Fig. S6. Therapeutic targeting of CMV in prostate cancer. Fig. S7. Mechanism of ellipticine and mithramycin A. Fig. S8. Maribavir in a prostate cancer xenograft model. Table S1. Characteristics of aciclovir epidemiology cohort. Table S2. Small interfering RNA sequences. Table S3. List of custom primer/probes used for qPCR. Table S4. List of custom primer/probes used for RT‐qPCR. [file MOL2-19-3035-s001.pdf]

Supporting information

Supplemental Fig. 1. CMV DNA and protein in the prostate
Supplemental Fig. 2. CMV in prostate cancer
Supplemental Fig. 3. CMV in prostate cancer metastases
Supplemental Fig. 4. CMV proteins are detected in prostate cancer cells and can be reduced with RNAi
Supplemental Fig. 5. Pre-clinical evaluation of aciclovir
Supplemental Fig. 6. Therapeutic targeting of CMV in prostate cancer
Supplemental Fig. 7. Mechanism of ellipticine and mithramycin A
Supplemental Fig. 8. Maribavir in a prostate cancer xenograft model

Table S1. Characteristics of aciclovir epidemiology cohort
Table S2. Small interfering RNA sequences
Table S3. List of custom primer/probes used for qPCR
Table S4. List of custom primer/probes used for RT-qPCR

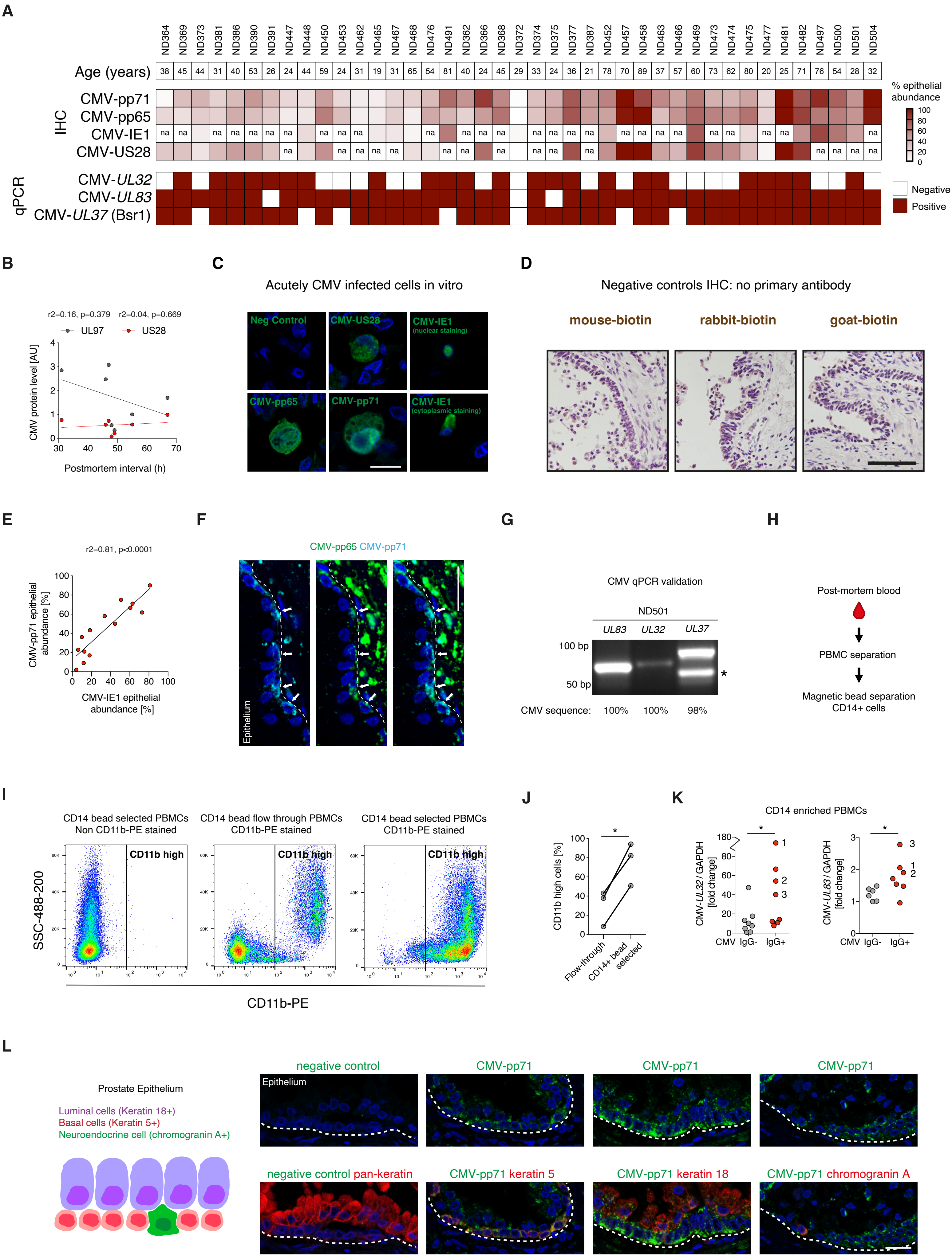
A
Age (years)
IHC
CMV-pp71
CMV-pp65
CMV-IE1
CMV-US28
qPCR
CMV-UL32
CMV-UL83
CMV-UL37 (Bsr1)
% epithelial abundance
Negative
Positive
B
r2=0.16, p=0.379 r2=0.04, p=0.669
UL97 US28
CMV protein level [AU]
Postmortem interval (h)
C
Acutely CMV infected cells in vitro
Neg Control
CMV-US28
CMV-IE1 (nuclear staining)
CMV-pp65
CMV-pp71
CMV-IE1 (cytoplasmic staining)
D
Negative controls IHC: no primary antibody
mouse-biotin
rabbit-biotin
goat-biotin
E
r2=0.81, p<0.0001
CMV-pp71 epithelial abundance [%]
CMV-IE1 epithelial abundance [%]
F
CMV-pp65 CMV-pp71
Epithelium
G
CMV qPCR validation
ND501
UL83 UL32 UL37
100 bp
50 bp
CMV sequence: 100% 100% 98%
H
Post-mortem blood
PBMC separation
Magnetic bead separation CD14+ cells
I
CD14 bead selected PBMCs Non CD11b-PE stained
CD14 bead flow through PBMCs CD11b-PE stained
CD14 bead selected PBMCs CD11b-PE stained
CD11b high
SSC-488-200
CD11b-PE
J
CD11b high cells [%]
Flow-through
CD14+ bead selected
K
CD14 enriched PBMCs
CMV-UL32 / GAPDH [fold change]
CMV-UL83 / GAPDH [fold change]
CMV IgG- IgG+
L
Prostate Epithelium
Luminal cells (Keratin 18+)
Basal cells (Keratin 5+)
Neuroendocrine cell (chromogranin A+)
negative control
CMV-pp71
Epithelium
negative control pan-keratin
CMV-pp71 keratin 5
CMV-pp71 keratin 18
CMV-pp71 chromogranin A

Supplemental Fig. 1: CMV DNA and protein in the prostate

A) Heat map to summarize CMV detection in postmortem donors. CMV epithelial abundance was analyzed with four CMV IHC assays detecting CMV-pp71, CMV-pp65, CMV-IE1 and CMV-US28 respectively. Heat map shows epithelial abundance of CMV in percentage. CMV DNA was examined with three CMV qPCR assays; CMV-UL32, CMV-UL83 and CMV-UL37 (DNA pre-treated with *bsr1*). Na is not analyzed. **B)** Linear regression comparing postmortem interval (time from death to tissue collection) with CMV-UL97 and CMV US28 protein in Fig. 1B, as quantified using B-actin as protein control. n=7, UL97: p=0.379; US28: p=0.669. **C)** IHC of acutely CMV infected cells *in vitro.* No staining was observed when primary antibody was omitted in negative (neg) control. CMV proteins were detected in cytoplasm and cell nuclei. Scale bar: 20 μm. Cell nuclei are labeled in blue with DAPI. **D)** CMV IHC assays with no primary antibody resulted in no DAB detection in prostate tissue. Three different secondary antibodies were used: mouse-biotin, rabbit-biotin or goat-biotin. Cell nuclei are labeled with hematoxylin in purple. Scale bar: 50 μm. **E)** Linear regression comparing CMV-pp71 epithelial abundance with CMV-IE1 epithelial abundance in prostate in percentage. P<0.0001. **F)** Co-labeling of CMV-pp65 (green) and CMV-pp71 (magenta). Arrows point to examples of epithelial cells positive for both proteins. Scale bar: 25 μm. Cell nuclei are labeled in blue with DAPI. Dotted line in depicts the basal lamina of the epithelium. **G)** Representative gel of prostate qPCR products with taqman primer/probes UL83, UL32 and UL37. For UL37 qPCR DNA was pre-treated with restriction enzyme bsr1. Sanger sequencing of cloned PCR products showed 100%, 100% and 98% match respectively to the merlin CMV genome. Asterisk point to CMV *UL37* PCR product. Bp = base pairs. **H)** Illustration of $CD14^+$ cell enrichment from blood in post-mortem donors. **I)** Bead enrichment of $CD14^+$ cells (which include monocytes) from peripheral blood mononuclear cells (PBMC) was validated with FACS. Cells were stained with a PE conjugated antibody against CD11b, which labels myeloid lineage cells. Unstained CD14 enriched cells (panel 1), CD11b stained CD14 bead flow through (panel 2) and CD11b stained CD14 enriched cells (panel 3) were examined. FACS plots show side scatter (SSC) on y-axes and CD11b-PE intensity on x-axes. The highest density of cells is shown in red, with decreasing density in yellow, green and blue. **J)** Percentage of CD11b high cells in CD11b stained CD14 bead flow through and CD11b stained CD14 enriched cells (n=3) were compared with paired t-test. **K)** qPCR of CMV-*UL32* and CMV-*UL83* in CD14 enriched PBMC DNA comparing CMV IgG^+ (n=8) and CMV IgG^- donors (n=8) with un-paired two-sided t-test. Data points 1, 2 and 3 are labeled in the two graphs for comparison. **L)** Illustration and staining of CMV-pp71 in three main cell types of the prostate epithelium; basal cells (Keratin 5^+), luminal cells (keratin 18^+), neuroendocrine cells (chromogranin A^+). Pan-keratin was used as a marker for all epithelial cells. Scale bar: 20 μm. Dotted line depicts the basal lamina of the

epithelium. Cell nuclei are labeled in blue with DAPI. $P < 0.05$: *. CMV = Cytomegalovirus. IHC = Immunohistochemistry. FACS = Fluorescence-activated cell sorting.

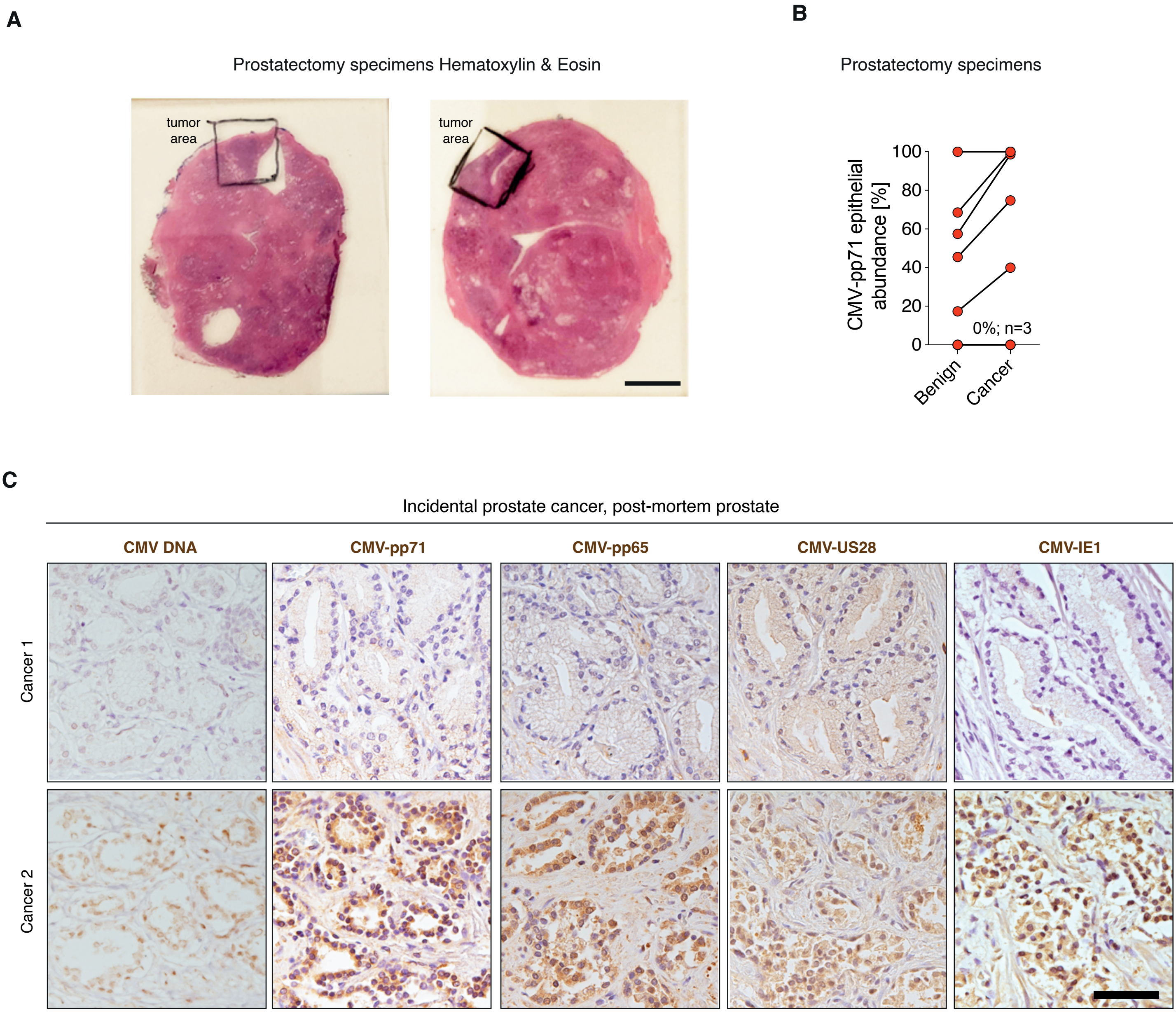
A
Prostatectomy specimens Hematoxylin & Eosin
tumor area
tumor area
B
Prostatectomy specimens
CMV-pp71 epithelial abundance [%]
100
80
60
40
20
0
0%; n=3
Benign
Cancer
C
Incidental prostate cancer, post-mortem prostate
CMV DNA
CMV-pp71
CMV-pp65
CMV-US28
CMV-IE1
Cancer 1
Cancer 2

Supplemental Fig. 2: CMV in prostate cancer

A) H&E of prostatectomy specimens. Scale bar: 1 cm. Tumor areas were annotated by a pathologist associated with PCBN. **B)** CMV-pp71 abundance (%) in matched benign and cancer epithelium in prostatectomy specimens (n=8). **C)** Prostate cancer in one post-mortem donor (Cancer 1) was negative and suspected prostate cancer in another post-mortem donor was positive (Cancer 2) for CMV by in situ hybridization (CMV DNA) and CMV IHC. Cell nuclei are labeled with hematoxylin. Scale bar: 100 µm.

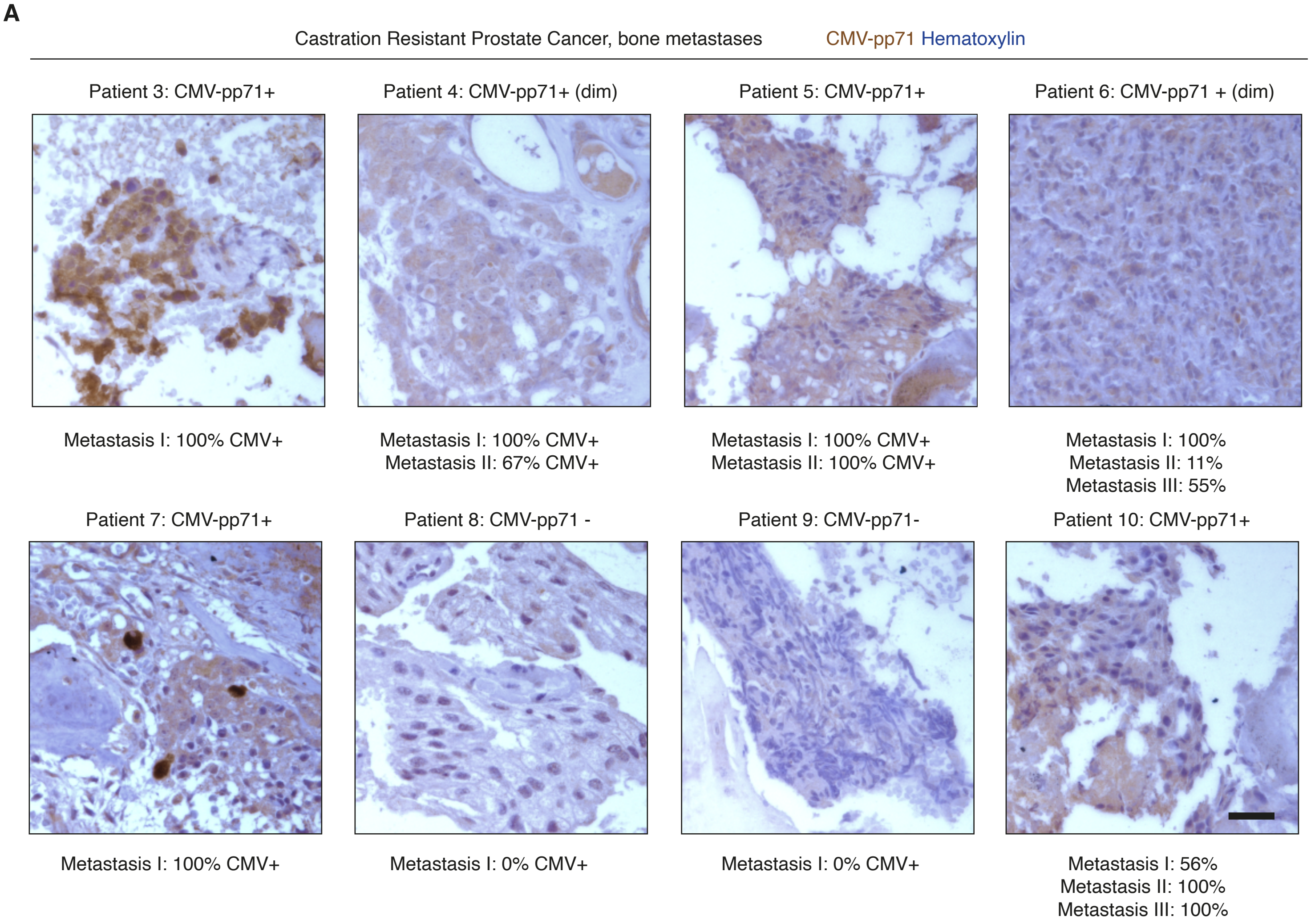
A
Castration Resistant Prostate Cancer, bone metastases
CMV-pp71 Hematoxylin
Patient 3: CMV-pp71+
Patient 4: CMV-pp71+ (dim)
Patient 5: CMV-pp71+
Patient 6: CMV-pp71 + (dim)
Metastasis I: 100% CMV+
Metastasis I: 100% CMV+
Metastasis II: 67% CMV+
Metastasis I: 100% CMV+
Metastasis II: 100% CMV+
Metastasis I: 100%
Metastasis II: 11%
Metastasis III: 55%
Patient 7: CMV-pp71+
Patient 8: CMV-pp71 -
Patient 9: CMV-pp71-
Patient 10: CMV-pp71+
Metastasis I: 100% CMV+
Metastasis I: 0% CMV+
Metastasis I: 0% CMV+
Metastasis I: 56%
Metastasis II: 100%
Metastasis III: 100%

Supplemental Fig. 3: CMV in prostate cancer metastases

Representative images of CMV-pp71 IHC in CRPC bone metastases. In five patients, more than one metastasis was examined, labelled metastases I, II and III and percentage of CMV-pp71+ areas are shown in figure. Brown is CMV-pp71 and violet is hematoxylin staining that label cell nuclei. Scale bar: 50 μm.

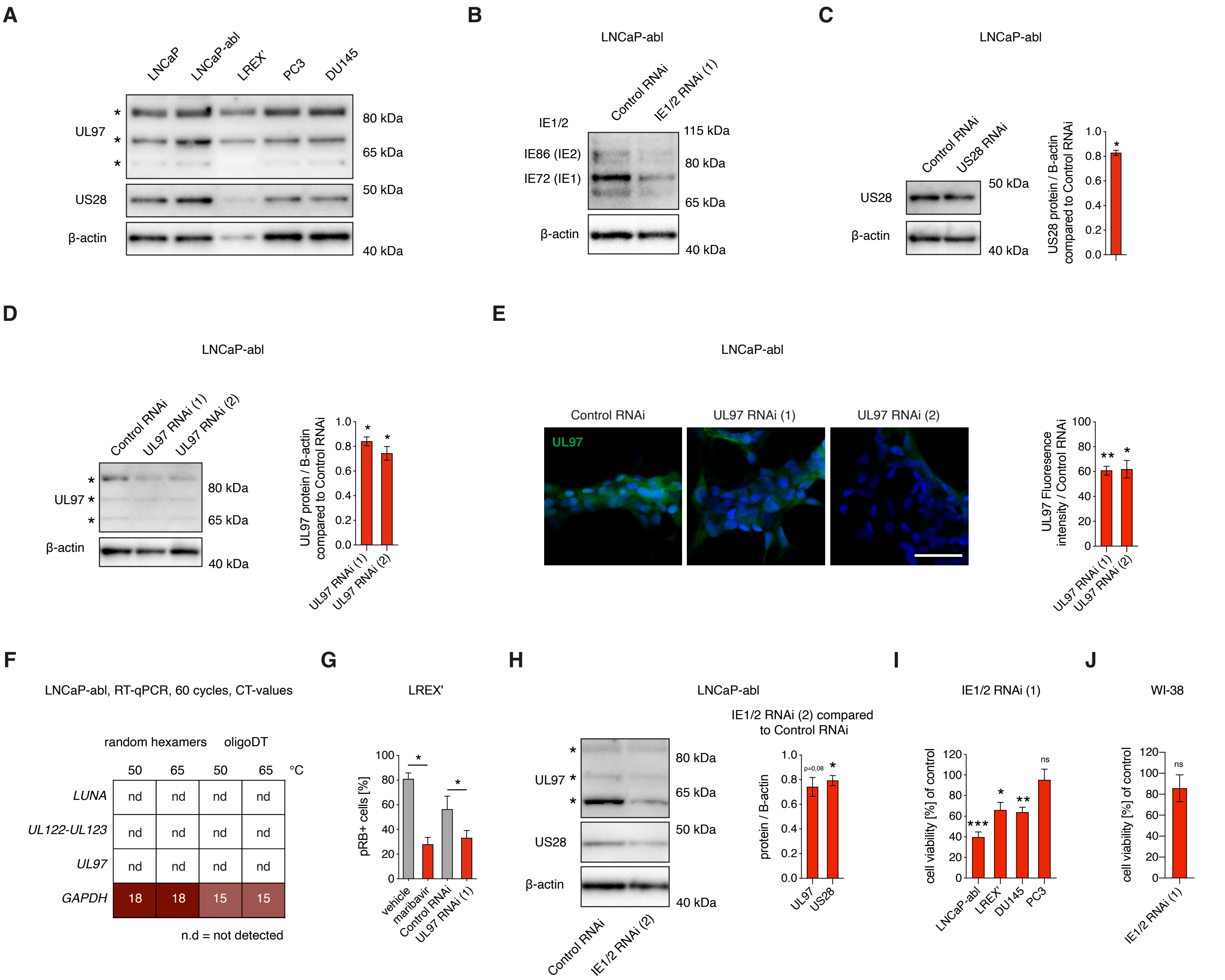
A
LNCaP
LNCaP-abl
LREX'
PC3
DU145
UL97
US28
β-actin
80 kDa
65 kDa
50 kDa
40 kDa
B
LNCaP-abl
Control RNAi
IE1/2 RNAi (1)
IE1/2
IE86 (IE2)
IE72 (IE1)
β-actin
115 kDa
80 kDa
65 kDa
40 kDa
C
LNCaP-abl
Control RNAi
US28 RNAi
US28
β-actin
50 kDa
40 kDa
US28 protein / B-actin compared to Control RNAi
D
LNCaP-abl
Control RNAi
UL97 RNAi (1)
UL97 RNAi (2)
UL97
β-actin
80 kDa
65 kDa
40 kDa
UL97 protein / B-actin compared to Control RNAi
E
LNCaP-abl
Control RNAi
UL97 RNAi (1)
UL97 RNAi (2)
UL97
UL97 Fluoresence intensity / Control RNAi
F
LNCaP-abl, RT-qPCR, 60 cycles, CT-values
random hexamers
oligoDT
50 65 50 65 °C
LUNA nd nd nd nd
UL122-UL123 nd nd nd nd
UL97 nd nd nd nd
GAPDH 18 18 15 15
n.d = not detected
G
LREX'
pRB+ cells [%]
vehicle
maribavir
Control RNAi
UL97 RNAi (1)
H
LNCaP-abl
UL97
US28
β-actin
80 kDa
65 kDa
50 kDa
40 kDa
Control RNAi
IE1/2 RNAi (2)
IE1/2 RNAi (2) compared to Control RNAi
protein / B-actin
p=0,08
UL97
US28
I
IE1/2 RNAi (1)
cell viability [%] of control
LNCaP-abl
LREX'
DU145
PC3
ns
J
WI-38
cell viability [%] of control
ns
IE1/2 RNAi (1)

Supplemental Fig. 4: CMV proteins are detected in prostate cancer cells and can be reduced with RNAi

A) Immunoblot of cell lines. CMV-US28, CMV-UL97 and β-actin expression were examined. Asterisks label three UL97 isoforms. **B)** Immunoblot of LNCaP-abl treated four days with control RNAi or IE1/2 RNAi (1). Note that the classical IE isoforms IE72 (IE1) and IE86 (IE2) were present and downregulated with IE1/2 RNAi (1) treatment. Experiment was performed in replicate. **C)** Immunoblot of LNCaP-abl four days after transfection with control RNAi or US28 RNAi. CMV-US28 and β-actin expression was examined and quantified (n=3). **D)** Immunoblot of LNCaP-abl four days after transfection with control RNAi, UL97 RNAi (1) or UL97 RNAi (2). CMV-UL97 and β-actin expression was examined and quantified (n=3). Asterisks label three UL97 isoforms. **E)** Mean UL97 fluorescence intensity in LNCaP-abl after three days transfection with control RNAi or UL97 RNAi (1). Scale bar 50 μm. Cell nuclei are labeled in blue with DAPI. **F)** Expression of the CMV genes *LUNA, UL122-UL123* and *UL97* was not detected in LNCaP-abl, independent of cDNA synthesis protocol (random hexamers or oligoDT) or cDNA synthesis temperature (50 or 65 ^{o}C). nd is not detected. **G)** Percentage of Ser^{807}/Ser^{811} pRB^{+} cells in LREX' three days after treatment with control RNAi or UL97 RNAi (1) and vehicle or 10 μM maribavir (n=3). **H)** Immunoblot of LNCaP-abl four days after transfection with control RNAi or IE1/2 RNAi (2). CMV-US28, CMV-UL97 and β-actin expression was examined and quantified (n=3). **I-J)** Cell viability in % compared to control, four days after treatment with control RNAi and IE1/2 RNAi (1) in prostate cancer cell lines (LNCaP-abl, LREX', n=5; DU145, n=4; PC3, n=3). **(I)** and three days after treatment in the fibroblast cell line WI-38 (n=3) **(J).** Data in bar graphs are shown as mean. Error bars in bar graphs are SEM. Treatments were compared with paired t-tests in (G) and with one-sample t-test in (C), (D), (E), (H) and (I-J). $p<0.05$ is*, $p<0.01$ is **, $p<0.001$ is ***. Ns is non-significant. kDa is kilodalton.

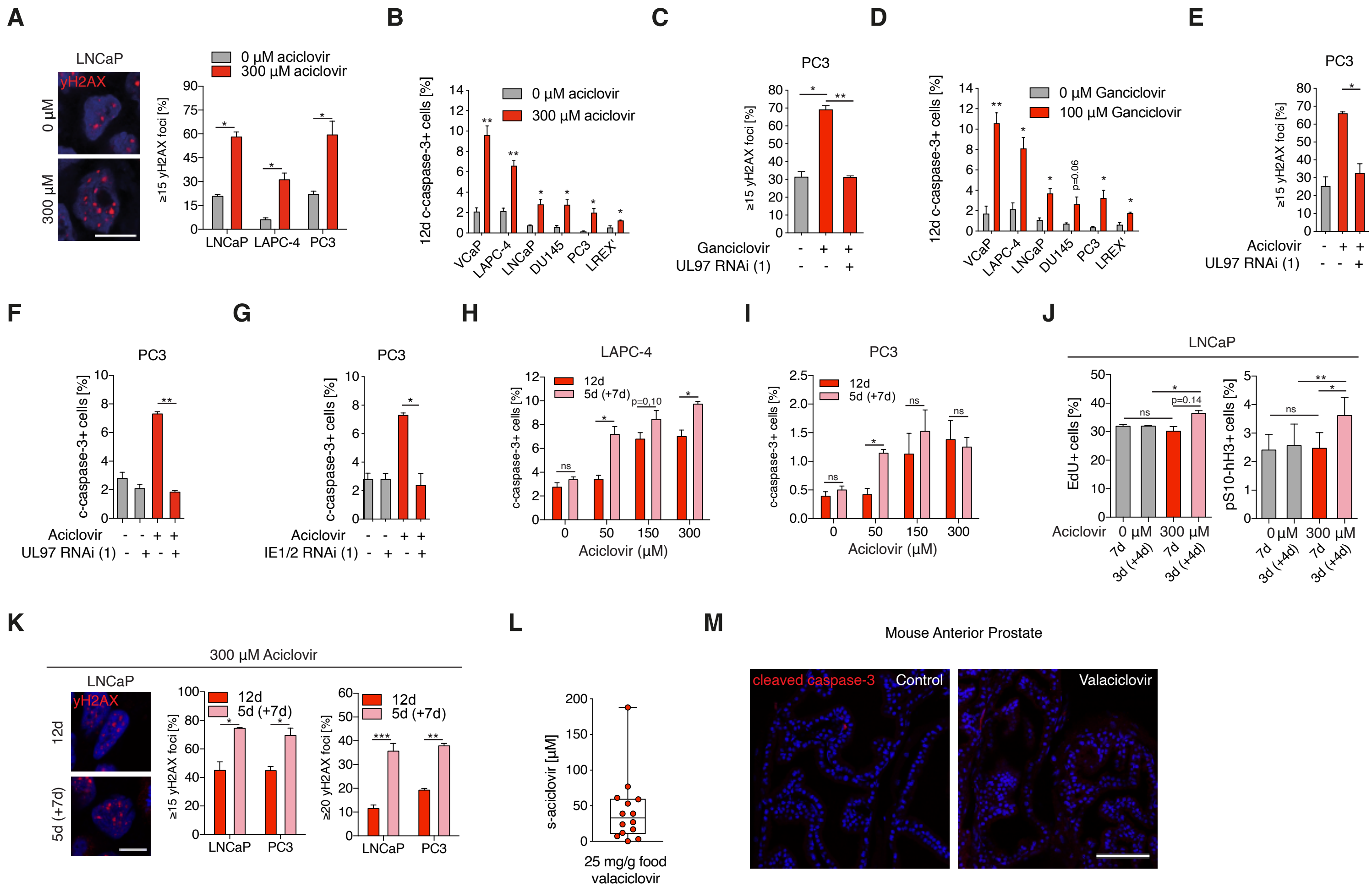

A
LNCaP
yH2AX
0 µM
300 µM
0 µM aciclovir
300 µM aciclovir
≥15 yH2AX foci [%]
LNCaP LAPC-4 PC3
B
12d c-caspase-3+ cells [%]
0 µM aciclovir
300 µM aciclovir
VCaP LAPC-4 LNCaP DU145 PC3 LREX'
C
PC3
≥15 yH2AX foci [%]
Ganciclovir
UL97 RNAi (1)
D
12d c-caspase-3+ cells [%]
0 µM Ganciclovir
100 µM Ganciclovir
p=0.06
VCaP LAPC-4 LNCaP DU145 PC3 LREX'
E
PC3
≥15 yH2AX foci [%]
Aciclovir
UL97 RNAi (1)
F
PC3
c-caspase-3+ cells [%]
Aciclovir
UL97 RNAi (1)
G
PC3
c-caspase-3+ cells [%]
Aciclovir
IE1/2 RNAi (1)
H
LAPC-4
c-caspase-3+ cells [%]
12d
5d (+7d)
p=0.10
Aciclovir (µM)
I
PC3
c-caspase-3+ cells [%]
12d
5d (+7d)
Aciclovir (µM)
J
LNCaP
EdU+ cells [%]
pS10-hH3+ cells [%]
p=0.14
Aciclovir 0 µM 300 µM
7d
3d (+4d)
K
300 µM Aciclovir
LNCaP
yH2AX
12d
5d (+7d)
≥15 yH2AX foci [%]
≥20 yH2AX foci [%]
LNCaP PC3
L
s-aciclovir [µM]
25 mg/g food valaciclovir
M
Mouse Anterior Prostate
cleaved caspase-3
Control
Valaciclovir

Supplemental Fig. 5: Pre-clinical evaluation of aciclovir

A) Aciclovir (300 μM) induced DNA damage in LNCaP, LAPC-4 and PC3, examined after 12 days by quantification of γH2AX foci per cell (n=3). Scale bar 20 μm. **B)** Aciclovir (300 μM) induced apoptosis (cleaved caspase-3) in cell lines examined after 12 days (VCaP, LAPC-4, LREX', n=3; LNCaP, DU145, PC3, n=4). **C)** In PC3, UL97 RNAi (1) reduced the ability of ganciclovir (GCV) to promote γH2AX foci (n=3), examined after 7 days. **D)** Ganciclovir (100 μM) induced apoptosis (cleaved caspase-3) in several cell lines examined after 12 days (VCaP, LAPC-4, LNCaP, LREX', n=3; DU145, n=4; PC3, n=5). **E)** In PC3, UL97 RNAi (1) reduced the ability of aciclovir (300 μM) to promote γH2AX foci (n=3), examined after 7 days. **F)** In PC3, UL97 RNAi (1) reduced the ability of aciclovir to induce apoptosis (n=3), examined after 7 days. **G)** In PC3, IE1/2 RNAi (1) reduced aciclovir induced apoptosis (n=3), examined after 7 days. **H-I)** LAPC-4 and PC3 treated with aciclovir in different concentrations continuously for 12 days or 5 days with and 7 days without (5d (+7d)) (n=3). **J)** LNCaP treated with aciclovir and treated with EdU 1 hour prior to fixation. Percentage of EdU^{+} cells and pS10-$hH3^{+}$ cells were quantified (n=3). **K)** Discontinuous aciclovir treatment increased γH2AX foci in LNCaP (n=4) and PC3 (n=3). Scale bar 20 μm. **L)** After seven to nine days of valaciclovir treatment, serum aciclovir levels were analyzed. Box plot (median, 25-75th percentiles, error bars show min-max values). **M)** In prostates of mice treated with valaciclovir 17-21 days, no induction of apoptosis (cleaved caspase-3^{+} cells) was observed. Scale bar: 100 μm. Cell nuclei are labeled in blue with DAPI. Data in bar graphs are shown as mean. Error bars in bar graphs are SEM. Data are analyzed with paired t-tests unless otherwise specified. $p<0.05$ is*, $p<0.01$ is **. $p<0.001$ is ***. Ns is non-significant. In (A), (C), (E) and (K), percentage of cells with 15 or 20 or more foci were analyzed and shown in graphs.

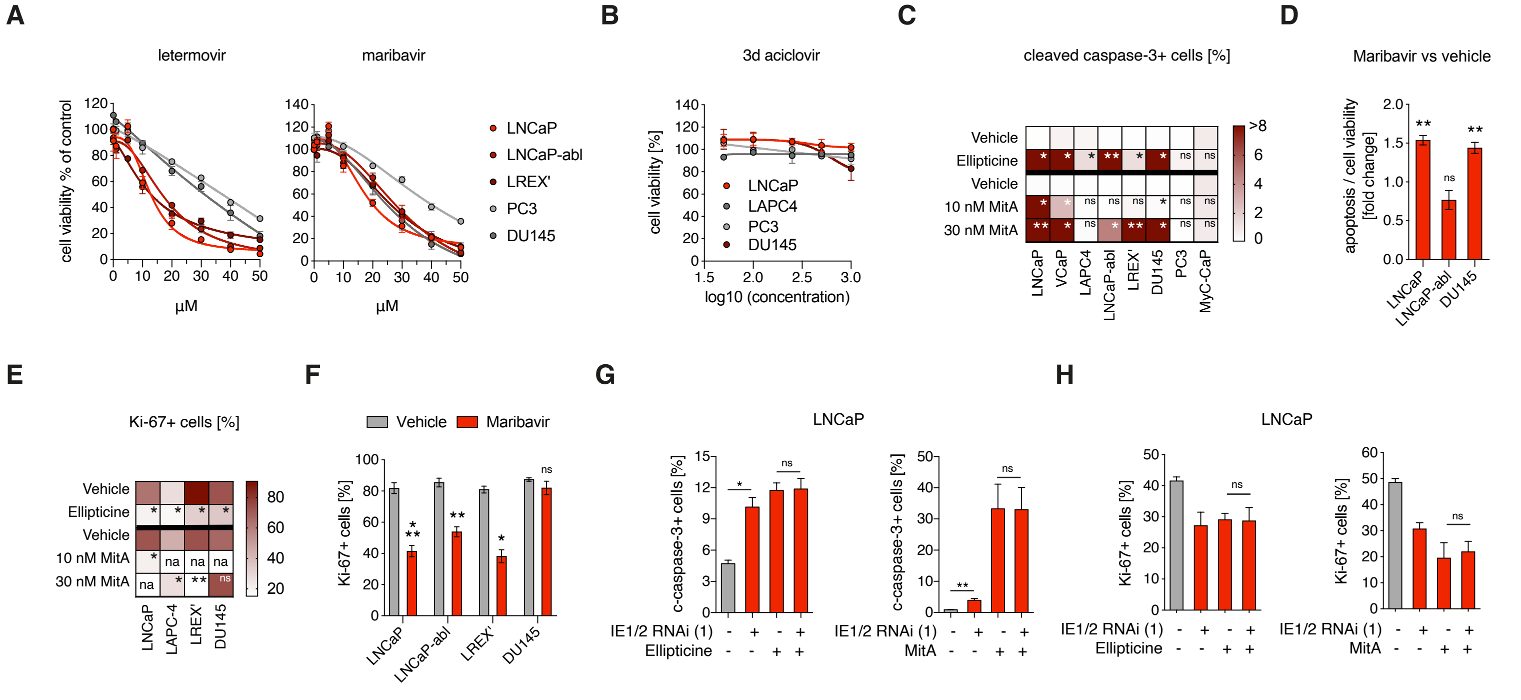
A
letermovir
maribavir
cell viability % of control
µM
LNCaP
LNCaP-abl
LREX'
PC3
DU145
B
3d aciclovir
cell viability [%]
log10 (concentration)
LNCaP
LAPC4
PC3
DU145
C
cleaved caspase-3+ cells [%]
Vehicle
Ellipticine
Vehicle
10 nM MitA
30 nM MitA
LNCaP
VCaP
LAPC4
LNCaP-abl
LREX'
DU145
PC3
MyC-CaP
D
Maribavir vs vehicle
apoptosis / cell viability [fold change]
LNCaP
LNCaP-abl
DU145
E
Ki-67+ cells [%]
Vehicle
Ellipticine
Vehicle
10 nM MitA
30 nM MitA
LNCaP
LAPC-4
LREX'
DU145
F
Vehicle
Maribavir
Ki-67+ cells [%]
LNCaP
LNCaP-abl
LREX'
DU145
G
LNCaP
c-caspase-3+ cells [%]
IE1/2 RNAi (1)
Ellipticine
MitA
H
LNCaP
Ki-67+ cells [%]
IE1/2 RNAi (1)
Ellipticine
MitA

Supplemental Fig. 6: Therapeutic targeting of CMV in prostate cancer

A) Cell viability examined after six days of treatment with letermovir (0-50 µM) or maribavir (0-50 µM) (n=3, except for LNCaP-abl, n=4). Lines show non-linear fit of treatment in each cell line. **B)** Cell viability in LNCaP, LAPC-4, PC3 and DU145 after three days of 0-1000 µM aciclovir (n=3), shown in log scale. **C)** Heat map showing mean of percentage cleaved caspase-3+ cells in cells treated with vehicle or 3 µM ellipticine (one day) (LNCaP, LAPC-4, LREX', PC3, n=5; VCaP, LNCaP-abl, DU145, MyC-CaP, n=3) and vehicle or 10-30 nM mithramycin A (three days) (LNCaP, DU145, n=4; VCaP, LAPC-4, LNCaP-abl, LREX', PC3, MyC-CaP, n=3). **D)** Cells were treated with vehicle or 30 µM maribavir and analyzed after three days for apoptosis, examined by the Caspase-Glo 3/7 assay, and cell viability (DU145, n=3; LNCaP, LNCaP-abl, n=4). Apoptosis compared to total viable cells was represented as fold change. **E)** Heat map showing mean of percentage Ki-67 in cells treated with vehicle or 3 µM ellipticine after one day (LNCaP, LAPC-4, n=3; LREX', DU145, n=4) and vehicle or 10-30 nM mithramycin A after three days (LNCaP, LAPC-4, LREX', n=3; DU145, n=4). Na is not analyzed. **F)** Cell lines treated with vehicle or 30 µM maribavir and analyzed after three days for percentage of Ki-67^{+} cells (n=3). **G-H)** LNCaP was transfected with Control RNAi or IE1/2 RNAi (1) four days in total and co-treated with ellipticine for one day and mithramycin A for three days (n=3). Percentage of cleaved caspase-3 and Ki-67^{+} cells are shown in bar graphs. Data in bar graphs are shown as mean. Error bars in bar graphs are SEM. Paired t-tests were performed in (C)-(H). $p<0.05$ is*, $p<0.01$ is **, $p<0.001$ is ***. Ns is non-significant. IE1/2 = Immediate early 1 and 2. MitA = Mithramycin A. RNAi = RNA interference.

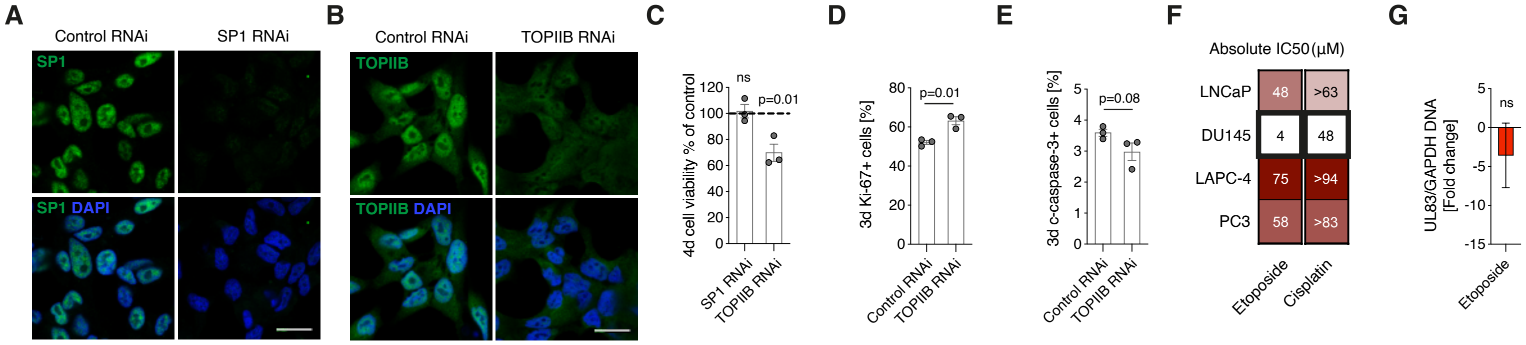
A
Control RNAi
SP1 RNAi
SP1
SP1 DAPI
B
Control RNAi
TOPIIB RNAi
TOPIIB
TOPIIB DAPI
C
4d cell viability % of control
ns
p=0.01
SP1 RNAi
TOPIIB RNAi
D
3d Ki-67+ cells [%]
p=0.01
Control RNAi
TOPIIB RNAi
E
3d c-caspase-3+ cells [%]
p=0.08
Control RNAi
TOPIIB RNAi
F
Absolute IC50 (µM)
LNCaP 48 >63
DU145 4 48
LAPC-4 75 >94
PC3 58 >83
Etoposide
Cisplatin
G
UL83/GAPDH DNA [Fold change]
ns
Etoposide

Supplemental Fig. 7: Mechanism of ellipticine and mithramycin A

A-B) SP1 and TOPIIB protein was reduced upon transfection with RNAi in LNCaP. Scale bar is 25 μm. **C)** Cell viability shown as percentage of control four days after transfection with SP1 RNAi or TOPIIB RNAi in LNCaP (n=3). **D-E)** Percentage of Ki-67+ cells and cleaved caspase-3^+ cells were evaluated in LNCaP transfected with Control RNAi or TOPIIB RNAi after three days (n=3). **F)** Heat map with absolute IC50 in cell lines treated with cisplatin (n=2) or etoposide (n=3) three days, Thick boxes show the most drug sensitive cell lines. Lighter color represent lower absolute IC50 and therefore higher sensitivity. **G)** CMV DNA abundance determined with CMV-*UL83* qPCR in DU145 four days after treatment with 3 μM etoposide compared to vehicle (n=3). Data is displayed as negative fold change. $p<0.05$ is*, $p<0.01$ is **. Ns is non-significant. CMV = Cytomegalovirus. IC50 = Half-maximal inhibitory concentration. RNAi = RNA interference.

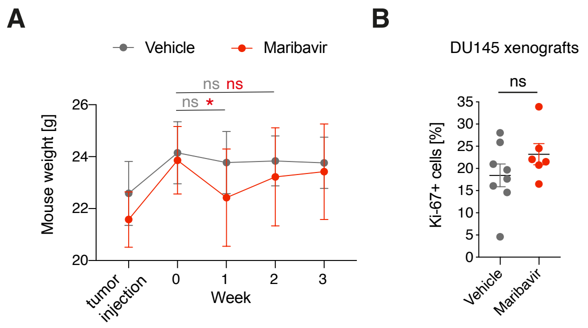
A
Vehicle
Maribavir
ns ns
ns *
Mouse weight [g]
26
24
22
20
tumor injection
0
1
2
3
Week
B
DU145 xenografts
ns
Ki-67+ cells [%]
35
30
25
20
15
10
5
0
Vehicle
Maribavir

Supplemental Fig. 8: Maribavir in a prostate cancer xenograft model

B) Animal weight was examined at time of tumor injection and weekly at start of treatment with vehicle (n=8) or maribavir (n=10). Error bars are SD. Unpaired two-tailed student's t-tests were performed comparing 0-1 weeks and 0-2 weeks for vehicle (grey) and maribavir (red) treated mice. **B)** Ki-67+ cells in tumors of mice treated with vehicle (n=8) or maribavir (n=6) 3.5 weeks. Line shows mean. Error bars are SEM. Unpaired two-tailed student's t-test was performed. $p<0.05$ is*, ns is non-significant.

Table S1.
Characteristics of aciclovir epidemiology cohort

	Aciclovir non-users<br>N (%)	Aciclovir users<br>N (%)
Overall	780,580 (100)	156,116 (100)
**Age at index date (years)**		
Mean (SD)	61.5 (12.7)	61.5 (12.7)
**Calendar year**		
1996-2000	94,130 (12.1)	18,826 (12.1)
2001-2005	132,945 (17.0)	26,589 (17.0)
2006-2010	167,505 (21.5)	33,501 (21.5)
2011-2015	186,550 (23.9)	37,310 (23.9)
2016-2020	199,450 (25.6)	39,890 (25.6)
**Charlson Comorbidity Index**		
Low (comorbidity score = 0)	550,099 (70.6)	88,786 (63.9)
Medium (comorbidity score 1 or 2)	176,268 (22.6)	39,992 (25.6)
High (comorbidity score 3+)	53,413 (6.8)	16,338 (10.5)

Table S2.

Small interfering RNA sequences

SP1	5’ GCAGACACAGCAGCAACAAAUUCUU ‘3
	5’ AAGAAUUUGUUGCUGCUGUGUCUGC ‘3
TOPIIB	5’ CCAGCAUGAUGAUAGUUCCUCCGAU ‘3
	5’ AUCGGAGGAACUAUCAUCAUGCUGG ‘3
CMV-IE1/2 (1)	5’ ACCUUUGAACAAGUGACCGAGGAUU ‘3
	5’ AAUCCUCGGUCACUUGUUCAAAGGU ‘3
CMV-IE1/2 (2)	5’ GGAAGGAGGUUAACAGUCAUU ‘3
	5’ UGACUGUUAACCUCCUUCCUU ‘3
CMV-UL97 (1)	5’ UCAGCGAGCCCUAUCCGGAUUACAA ‘3
	5’ UUGUAAUCCGGAUAGGGCUCGCUGA ‘3
CMV-UL97 (2)	5’ AAAGCAGGGUGGUAACAUUCGCGCA ‘3
	5’ UGCGCGAAUGUUACCACCCUGCUUU ‘3
CMV-US28	5’ AGGUCGCAUUGUACGGGUATT ‘3
	5’ UACCCGUACAAUGCGACCUTT ‘3

Table S3.

List of custom primer/probes used for qPCR

UL32	F: GGCGCGGGAACCTCTT
	R: CCGTGGGCGACAAAACG
	Probe: CAGCCGTCAGCCTCG
UL36	F: GAAAGAAGGGACACCGAAACCA
	R: GACAGGTGGGTGTCTTTTCCA
	Probe: ACGCACGATGGCCTC
UL37 (X1)	F: GTGGCCGCGCTCTTG
	R: GCTCTGTGTCCTCCGTTACG
	Probe: CCTCCCCGGCCTCG
UL38	F: CGCTCCCACGTCCGT
	R: AGCTGGTGGAAGACCATCAC
	Probe: CAGCACGCGCACACTA
UL83	F: CCCAGCGTGACGTGCATAA
	R: AGGTGTACCTGGAGTCCTTCTG
	Probe: CTCCGGCAAGCTCT
UL87	F: GTGCTGTTTCTGCGTGCTT
	R: CAACTGCAGCCGCTTCTC
	Probe: TCGACCGTGCAGCTTG
UL97	F: ACCGTCTGCGCGAATGTTA
	R: GTCGCAGATGAGCAGCTTCT
	Probe: CCACCCTGCTTTCCG
UL122	F: GCTTGATGTCTTCCTGTTTGATGAG
	R: ACGCGTCCTTTCAAGGTGATTATTA
	Probe: CCTCCCGCGCCTATC

Table S4.

List of custom primer/probes used for RT-qPCR

LUNA	F: CCTCGGTGGGTGGTAATCC
	R: GCGCCGTCTCCGAGTTT
	Probe: CTCCCGCAGTCCCC
UL97	F: ACCGTCTGCGCGAATGTTA
	R: GTCGCAGATGAGCAGCTTCT
	Probe: CCACCCTGCTTTCCG
UL122-UL123	Forward: TGACGAGGGCCCTTCCT
	Reverse: CCTTGGTCACGGGTGTCT
	Probe: AAGGTGCCACGGCCCG
AR-V7	Forward: TGTCGTCTTCGGAAATGTTATGA
	Reverse: TCATTTTGAGATGCTTGCAATTG
	Probe: TCTGGGAGAAAAATT
